# MiRNA-10b Reciprocally Stimulates Osteogenesis and Inhibits Adipogenesis Partly through the TGF-β/SMAD2 Signaling Pathway

**DOI:** 10.14336/AD.2018.0214

**Published:** 2018-12-04

**Authors:** Hongling Li, Junfen Fan, Linyuan Fan, Tangping Li, Yanlei Yang, Haoying Xu, Luchan Deng, Jing Li, Tao Li, Xisheng Weng, Shihua Wang, Robert Chunhua Zhao

**Affiliations:** ^1^Institute of Basic Medical Sciences Chinese Academy of Medical Sciences, School of Basic Medicine Peking Union Medical College, Center of Excellence in Tissue Engineering Chinese Academy of Medical Sciences, Beijing Key Laboratory (No. BZO381), Beijing 100005, China.; ^2^Department of Orthopaedic Surgery, Peking Union Medical College Hospital, Peking Union Medical College, Beijing 100730, China.; ^3^Current address: Department of Orthopaedic Surgery, The Affiliated Hospital of Qingdao University, Qingdao 266003, China.

**Keywords:** mesenchymal stem cells, miR-10b, osteogenesis, adipogenesis, SMAD2

## Abstract

As the population ages, the medical and socioeconomic impact of age-related bone disorders will further increase. An imbalance between osteogenesis and adipogenesis of mesenchymal stem cells (MSCs) can lead to various bone and metabolic diseases such as osteoporosis. Thus, understanding the molecular mechanisms underlying MSC osteogenic and adipogenic differentiation is important for the discovery of novel therapeutic paradigms for these diseases. miR-10b has been widely reported in tumorigenesis, cancer invasion and metastasis. However, the effects and potential mechanisms of miR-10b in the regulation of MSC adipogenic and osteogenic differentiation have not been explored. In this study, we found that the expression of miR-10b was positively correlated with bone formation marker genes ALP, RUNX2 and OPN, and negatively correlated with adipogenic markers CEBPα, PPARγ and AP2 in clinical osteoporosis samples. Overexpression of miR-10b enhanced osteogenic differentiation and inhibited adipogenic differentiation of human adipose-derived mesenchymal stem cells (hADSCs) *in vitro*, whereas downregulation of miR-10b reversed these effects. Furthermore, miR-10b promoted ectopic bone formation *in vivo*. Target prediction and dual luciferase reporter assays identified SMAD2 as a potential target of miR-10b. Silencing endogenous SMAD2 expression in hADSCs enhanced osteogenesis but repressed adipogenesis. Pathway analysis indicated that miR-10b promotes osteogenic differentiation and bone formation via the TGF-β signaling pathway, while suppressing adipogenic differentiation may be primarily mediated by other pathways. Taken together, our findings imply that miR-10b acts as a critical regulator for balancing osteogenic and adipogenic differentiation of hADSCs by repressing SMAD2 and partly through the TGF-β pathway. Our study suggests that miR-10b is a novel target for controlling bone and metabolic diseases.

The global population is aging at an unprecedented rate, and thus, the prevalence of chronic age-related diseases is inevitably increasing, which will have a profound impact on health care systems [1]. Among bone-related disorders, osteoporosis is one of the most common orthopedic problems in aged populations [2]. Osteoporosis is characterized by decreased bone mass and bone tissue micro-architectural deterioration [3]. Most patients show an increase in marrow fat content accompanied by bone loss [4]. Mesenchymal stem cells (MSCs) are one of the most important types of adult stem cells and are the common progenitors of osteoblasts and adipocytes [5]. Therefore, a reciprocal inhibitory relationship exists between osteogenic and adipogenic lineage commitment and differentiation. Inducers of adipocyte differentiation may inhibit cell differentiation into osteoblasts, and vice versa [6-9]. As the average age of the population grows, an increasing number of people are living with osteoporosis and suffer from fractures and other skeletal disabilities [10]. Thus, a clear understanding of the molecular mechanisms governing the balance between osteogenic and adipogenic differentiation of MSCs is of great significance to elucidate the pathogenesis of bone and metabolic diseases, and to develop novel and effective therapies.

In modern molecular biology, the discovery of small, endogenous, single-stranded noncoding microRNAs (miRNAs) and their roles in post-transcriptional gene regulation has been a significant scientific advancement [11]. Recent studies revealed that miRNAs play crucial roles in various biological processes, including cellular differentiation, proliferation, apoptosis, and tissue development [12-15]. The function of miRNAs in bone formation and adipogenesis is now emerging. Several miRNAs including miR-196a [16], miR-335-5p [17], miR-216a [18], miR-26a [19] and miR-133 [20] can regulate osteogenesis, and miR-143 [21], miR-155 [22], miR-30 and miR-642a-3p [23] are modulators of adipogenesis. However, only a few miRNAs have been implicated to be responsible for both processes, such as miR-204 [24], miR-17-5p, miR-106a [25], miR-30e [26] and miR-194 [27]. It suggests that adipocyte and osteoblast differentiation are tightly regulated by specific miRNAs in hMSCs.

miR-10b is a well-known oncogenic miRNA and can promote growth and metastasis of cancer cells. Abnormal expression of miR-10b is indicative of poor prognosis in various types of cancer such as breast cancer [28], melanoma [29], pancreatic cancer [30], hepatocellular carcinoma [31], colorectal cancer [32], lung cancer [33] and ovarian cancer [34]. However, the effects and potential mechanisms of miR-10b in the regulation of MSC adipogenic and osteogenic differentiation have not been explored.

In this study, we revealed that the expression of miR-10b was positively correlated with bone formation marker genes and negatively correlated with adipocyte formation markers in clinical osteoporosis samples. miR-10b can promote osteogenesis *in vitro*, enhance bone formation *in vivo*, and repress adipogenesis by targeting SMAD2 and partly through the TGF-β signaling pathway. Our findings suggest that miR-10b may serve as a novel therapeutic agent for the prevention and treatment of osteoporosis and other bone metabolism-related diseases.

## MATERIALS AND METHODS

### Human adipose-derived mesenchymal stem cells (hADSCs) isolation and culture

hADSCs were isolated from adipose tissue, which was collected from healthy women who underwent liposuction surgery. This procedure was approved by the Ethics Committee of the Chinese Academy of Medical Sciences and Peking Union Medical College. The isolation and culture procedure of hADSCs was described in previous studies [35]. Cells were cultured at 37 °C in a humidified incubator with 5% CO_2_. hADSCs in the third passage were used for the following experiments.

### Osteogenic and adipogenic differentiation of hADSCs *in vitro*

hADSCs were plated at a cell density of 2×10^5^ cells in six-well plates. At 80% confluence, the growth medium was replaced with osteoblast-specific induction medium containing high glucose of Dulbecco’s modified Eagle’s medium (H-DMEM) supplemented with 10% fetal bovine serum (Gibco, Carlsbad, CA, USA), 10 mM β-glycerophosphate (Sigma-Aldrich, St. Louis, MO, USA), 0.5 mM L-ascorbic acid (Sigma-Aldrich) and 0.01 mM dexamethasone (Sigma-Aldrich). For adipogenic differentiation, cells at 90% confluence were induced in adipogenic medium composed of H-DMEM supplemented with 10% fetal bovine serum, 1 μM dexamethasone, 0.5 mM isobutylmethylxanthine (Sigma-Aldrich) and 1 mM L-ascorbic acid.

### Clinical bone specimen preparation

Thirty osteoporosis patients who had a fracture caused by falling without obvious violence were enrolled in our study, and clinical bone specimens were collected from the Orthopaedic Department of Peking Union Medical College Hospital. None of the participants had a history of other diseases including metabolic or endocrine disease, chronic renal failure, chronic liver disease, malignancies or had received hormone replacement therapy. All clinical procedures were approved by the Ethics Committee of the Chinese Academy of Medical Sciences and Peking Union Medical College.

### Alkaline phosphatase and Alizarin red staining

Alkaline phosphatase (ALP) staining was performed using an ALP staining kit (Institute of Hematology and Blood Diseases Hospital, Chinese Academy of Medical Sciences, Tianjin, China) according to the manufacturer’s protocol. Alizarin red staining was performed to detect matrix mineralization deposition during the later stage of osteogenesis. Briefly, cells were washed twice with PBS, fixed in 4% paraformaldehyde for 10 min, rinsed with distilled water and stained with 1% Alizarin red (Leagene, Beijing, China) with pH 4.2 for 30 min at room temperature. Then, cells were rinsed with distilled water to remove the unbound dye and matrix calcification was shown with red deposition.

### ALP activity assay

The cells were washed three times with cold PBS and lysed in radioimmunoprecipitation (RIPA) lysis buffer (Beyotime, Shanghai, China). After sonication and centrifugation, the ALP activity in the supernatant was measured photometrically using the Alkaline Phosphatase Yellow Liquid Substrate System (pNPP, Sigma-Aldrich). In brief, 5 μl of the cell supernatant was incubated with 200 μl pNPP reagent at 37 °C for 30 min. The reaction was blocked by adding 50 μl of 3 M NaOH. Final absorbance was measured at 405 nm. The ALP activity was normalized to the total protein of the cell lysates.

### Oil red O staining and extraction

The cells in 24-well plates were washed twice with PBS and fixed with 4% paraformaldehyde for 10 min. The cells were then stained with filtered oil red O solution (stock solution: 1 mg/ml in isopropanol; working solution: 60% oil red O stock solution mixed with 40% distilled water) at 37 °C for 30 min. The cells were rinsed with distilled water to remove the unbound dye and were subsequently photographed. The dye of oil red O-positive cells was extracted by isopropanol, and the OD value was quantified at 510 nm wavelength by an ELISA microplate reader.

### Lentiviral vector preparation and infection

Lentivirus production was carried out according to protocols from GenePharma (www.genepharma.com/). The engineered pre-miRNA sequence was cloned into the lentiviral vector LV3-pGLV-H1-GFP+Puro. A lentiviral vector that expressed a scrambled RNA was used as a negative control (NC). The lentiviruses were produced through the transient transfection of 293T cells using Lipofectamine Plus, lentivirus vectors, and packaging plasmid mix. The packaged lentiviruses were named lenti-10b and lenti-NC, respectively. Cells were infected with lenti-10b or lenti-NC and GFP-positive cells were screened by puromycin.

### miRNA mimic, inhibitor and siRNA transfection

Third-passage hADSCs were transfected with miRNA inhibitor or siRNA by Lipofectamine 2000 (Invitrogen, Waltham, MA, USA) according to the manufacturer’s instructions. After transfection, the medium was replaced with regular growth medium or induction medium for differentiation. The miR-10b mimic, inhibitor and negative control were synthesized by Invitrogen. The siRNAs were obtained from GenePharma (Shanghai, China). The sequences of SMAD2 siRNA are listed in Supplementary Table 1.

### RNA isolation and quantitative real-time polymerase chain reaction (qRT-PCR) analysis

Total RNA was extracted from cultured cells or fresh bone tissues with TRIzol reagent (Invitrogen) and then treated with DNase I (Ambion, Austin, TX, USA) at 37 °C for 30 min. Reverse transcription was performed by using a Reverse Transcription kit (Takara, Japan) according to the manufacturer’s instructions. qRT-PCR was amplified with SYBR premix Ex Taq (Takara) using a Step One Plus Real-Time PCR Detection System (Applied Biosystems, Foster City, CA, USA). The relative expression of mRNA or miRNA was evaluated by the 2^-ΔΔCt^ method and normalized to the expression of GAPDH or U6, respectively. The primers used in this study are listed in Supplementary Table 1.

### Western blot analysis

Cells were washed three times with cold PBS. Protein was extracted with RIPA lysis buffer with PMSF (Beyotime) and quantified using a BCA Protein Assay kit (Beyotime). Western blot was performed as previously described [36]. Protein fractions were separated by 10% sodium dodecyl sulfate-polyacrylamide electrophoresis (SDS-PAGE) gel, and then transferred to 0.45 µm PVDF membranes (Millipore, Billerica, MA, USA). After blocking in 5% non-fat milk in TBST for 1 h, the membranes were incubated with specific primary antibodies at 4 °C overnight. Then secondary horseradish peroxidase-conjugated antibodies (Neobioscience, Shenzhen, China) were added, and the membranes were incubated at room temperature for 1 h. Immunodetection was visualized using a chemiluminescent ECL reagent (Millipore, USA).

### Ectopic bone formation in vivo 

All animal experiments were performed in accordance with guidelines and permissions of the Ethics Committee of the Chinese Academy of Medical Sciences and Peking Union Medical College. Approximately 2×10^6^ cells were mixed with wetted hydroxyapatite/tricalcium phosphate (HA/TCP) ceramic powder (80 mg; National Engineering Research Center for Biomaterials, Chengdu, China), incubated at 37 °C overnight and implanted subcutaneously into the dorsal surface of 8-week-old NOD/SCID mice, as previously described [37]. The implants were harvested after 8 weeks, fixed in 4% paraformaldehyde, decalcified in 10% EDTA and embedded in paraffin. Thin sections (5 μm) were stained with hematoxylin and eosin (H&E), masson trichrome, safranin O and immunohistochemical antibodies.

### Dual luciferase reporter gene construct and assay

Approximately 90 bp synthetic fragments of NFAT5, ESRRG, SMAD2, SMURF1 and HDAC4 3’UTR containing the predicted seed match site with miR-10b, or the corresponding mutated 3’UTR were inserted between the Not I and Xho I cleavage sites of the psiCHECK-2 vector (Promega, Madison, WI, USA), downstream of the Renilla luciferase reporter gene. The sequences of relative fragments are listed in Supplementary Table 2. The luciferase reporter vectors were co-transfected with 50 nM miR-10b mimic or miR-NC into 293T cells. Luciferase activity was measured in triplicate at 48 h after transfection using a Dual Luciferase Reporter Assay System (Promega). Renilla luciferase activity was normalized to firefly luciferase activity.

**Figure 1.**
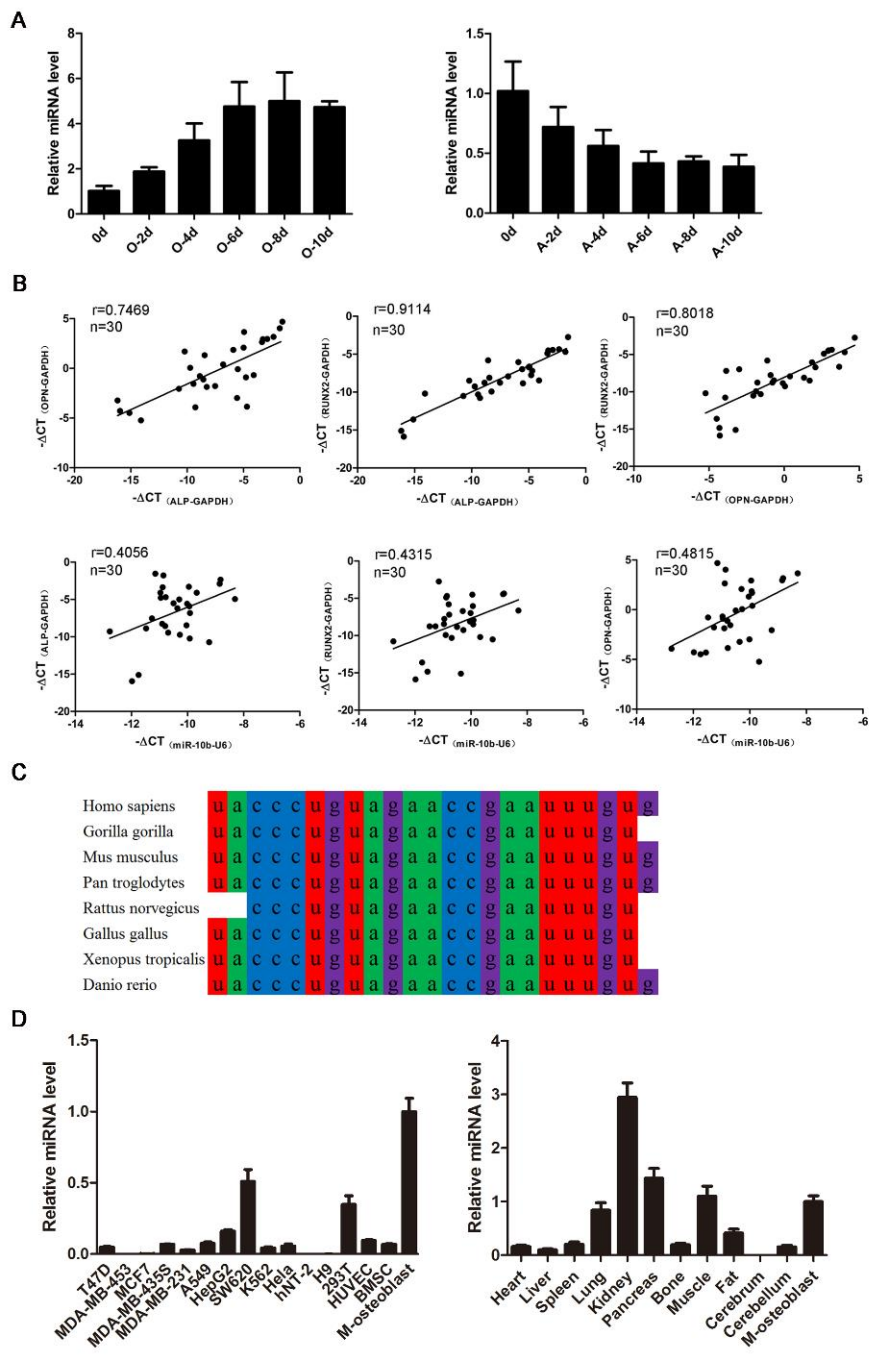
Expression pattern of miR-10b. (A) The dynamic expression of miR-10b during osteogenic and adipogenic differentiation of hADSCs. (B) The correlation between the expression of osteogenic-related genes and miR-10b in clinical osteoporosis samples. (C) The conservation of miR-10b in mammals. (D) miR-10b expression levels in various cell lines and mouse tissues (M-osteoblast: hADSC-derived osteoblast) were detected by qRT-PCR. The data, normalized to GAPDH or U6, are averages of 3 independent experiments (mean ± SD).

**Figure 2.**
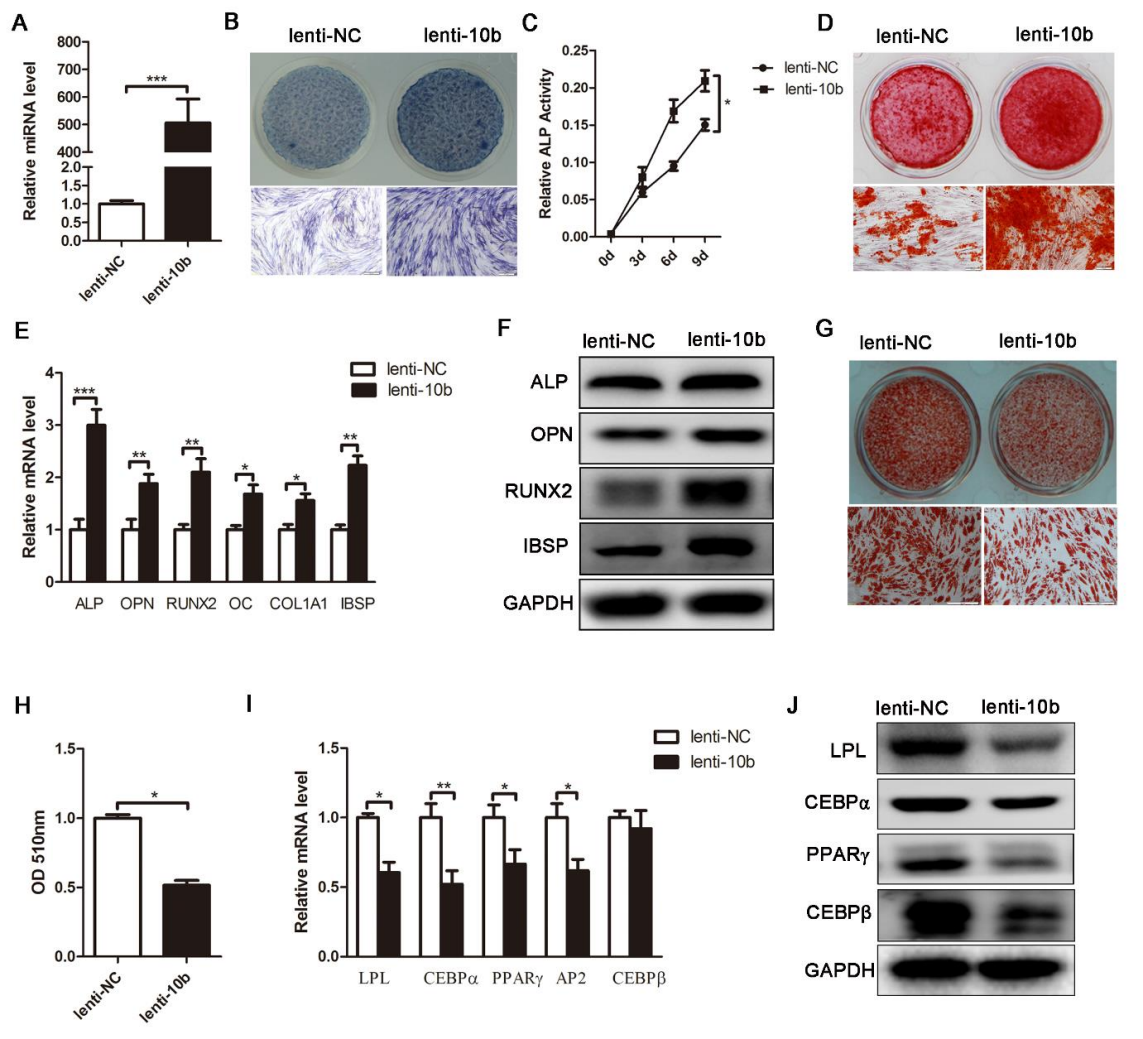
MiR-10b promotes osteogenic differentiation and inhibits adipogenic differentiation of hADSCs. (A) The miR-10b-expressing lentivirus increased the expression of mature miR-10b in hADSCs, analyzed by stem-loop qRT-PCR. (B and C) ALP staining was performed on day 4 and ALP activity was detected during osteogenic differentiation. (D) Alizarin red staining was performed to indicate mineral deposition on day 12. (E and F) Lenti-10b increased the mRNA and protein expression levels of osteogenic-specific markers on day 6 of osteogenic differentiation. (G) Oil red O staining was performed to detect the lipid droplets formation on day 10 of adipogenic differentiation. (H) The dye of oil red O-positive cells was extracted by isopropanol, and the OD value was quantified at 510 nm wavelength. (I and J) Lenti-10b decreased the mRNA and protein expression levels of adipogenic-specific markers. The data, normalized to U6 or GAPDH, are averages of 3 independent experiments (mean ± SD). ^*^P<0.05; ^**^P<0.01; ^***^P<0.001 compared with the control. Scale bars: 200 μm.

### Statistical analysis

The data were analyzed by using GraphPad Prism5 software (GraphPad Software Inc.). Data between two groups were analyzed using *t*-tests, and among multiple groups were assessed by one-way ANOVA. Differences were considered statistically significant at ^*^P<0.05, ^**^P<0.01 and ^***^P<0.001.

## RESULTS

### MiR-10b expression patterns during osteogenic differentiation and in clinical osteoporosis samples

To identify differentially expressed miRNAs during osteoblastic and adipocytic differentiation, a microarray analysis of miRNAs was performed to compare differentiated and undifferentiated hADSCs. According to fold change and *P*-values, we found that the expression of miR-10b increased during osteogenic differentiation but decreased during adipogenic differentiation (data not shown). The expression levels of miR-10b were confirmed at different time points during osteoblast and adipocyte differentiation by miRNA specific stem-loop RT-PCR. The results showed that miR-10b increased during osteogenic differentiation but decreased during adipogenic differentiation (Fig. 1A), suggesting that miR-10b is involved in regulating the balance of adipogenesis and osteogenesis of hADSCs. We collected 30 clinical samples from osteoporosis patients of different ages and analyzed the correlation between the expression of miR-10b and the bone formation marker genes ALP, OPN and RUNX2, and the correlation between miR-10b and adipocyte formation markers CEBPα, PPARγ and AP2. We found that the expression levels of ALP, OPN and RUNX2 were positively correlated with each other and the expression of miR-10b was also positively correlated with these bone formation-related genes (Fig. 1B), however, miR-10b was negatively correlated with adipogenic markers CEBPα, PPARγ and AP2 (Supplementary Fig. 1), implying miR-10b might play an important role in osteoporosis. Sequence analysis of miR-10b revealed that it is highly conserved across species, including Homo sapiens, Gorilla gorilla, Mus musculus, Pan troglodytes, Rattus norvegicus, Gallus gallus, Xenopus tropicalis and Danio rerio (Fig. 1C). In addition, we analyzed the expression of miR-10b in various cell lines and mouse tissues. miR-10b exhibited relatively low expression levels in several cell lines (e.g., breast cancer cell line, HeLa and H9), whereas it was highly expressed in hADSC-derived osteoblasts. miR-10b expression levels in the lung, kidney, pancreas, muscle and osteoblasts were also much higher than that in other tissues (Fig. 1D).

**Figure 3.**
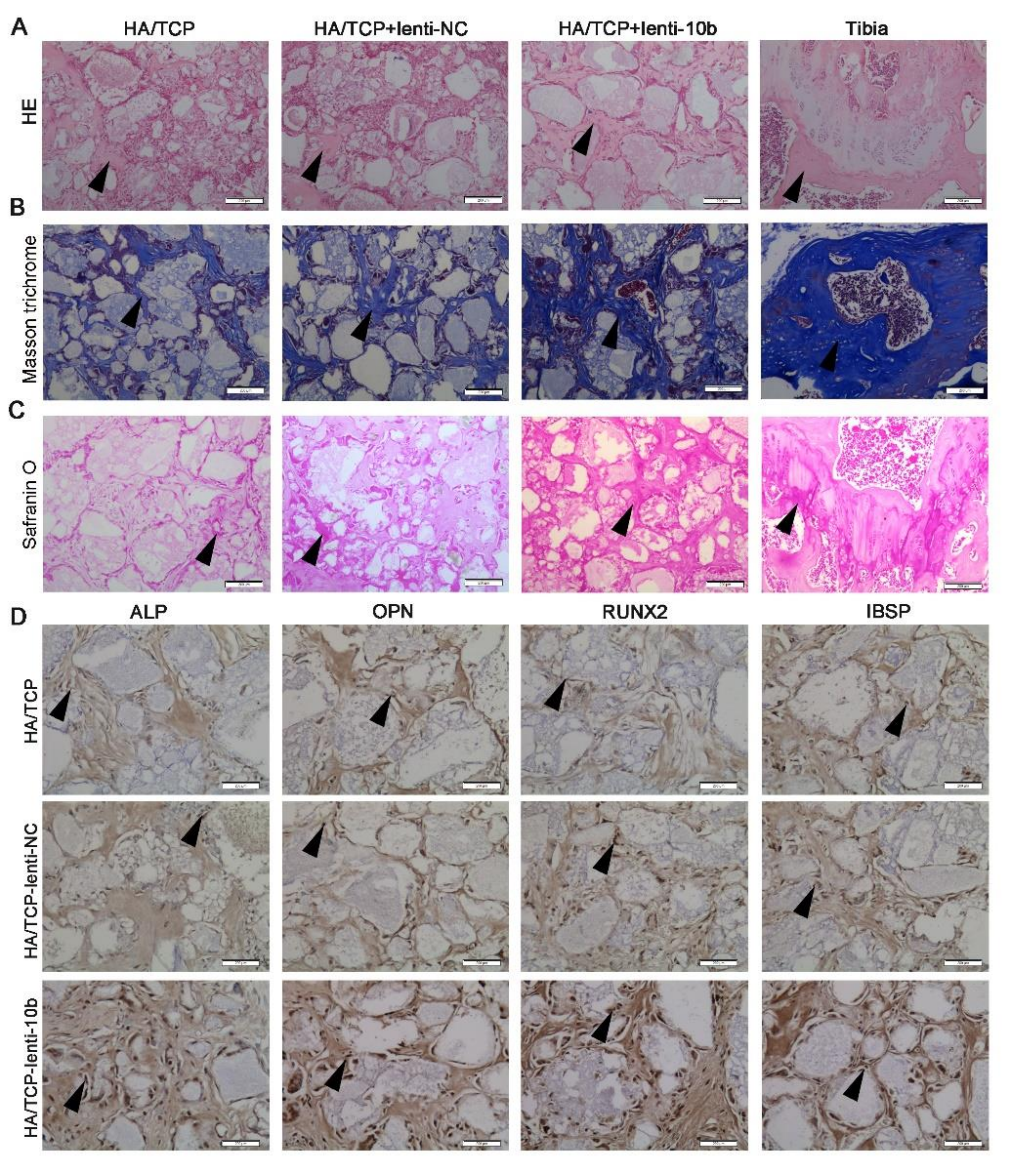
MiR-10b promotes the ectopic bone formation of hADSCs *in vivo*. (A) H&E staining was used to analyze osteoid formation in the xenografts. (B) Masson trichrome staining indicated collagen. (C) Safranin O staining revealed cartilage formation in the xenografts. (D) Immunohistochemical staining showed the expression levels of osteogenic markers in the xenografts. Black arrows represent positive signals. Scale bars: 200 μm.

**Figure 4.**
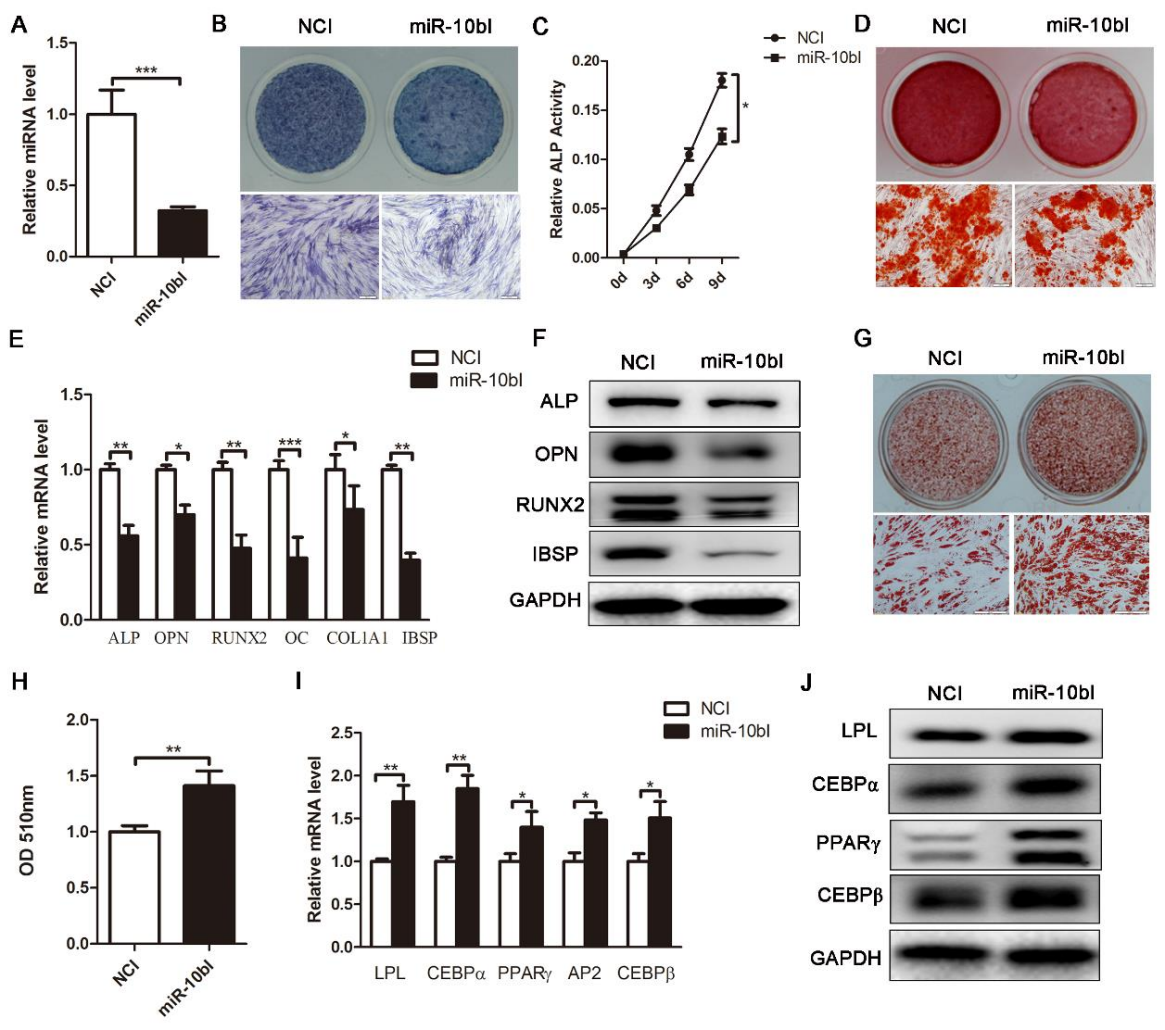
Downregulation of endogenous miR-10b suppresses osteogenic differentiation and enhances adipogenic differentiation. (A) miR-10b expression was determined by stem-loop RT-PCR. (B and C) ALP staining and ALP activity were performed after inhibiting miR-10b. (D) Alizarin red staining was performed on day 12. (E and F) qRT-PCR and western blot were performed to analyze the mRNA and protein levels of osteogenic-specific markers after miR-10b inhibitor transfection. (G and H) Oil red O staining and extraction were performed to detect the formation of lipid droplets on day 10 of adipogenic differentiation. (I and J) The expression of adipogenic-specific markers was analyzed by qRT-PCR and western blot. The data, normalized to GAPDH, are averages of 3 independent experiments (mean ± SD). ^*^P<0.05; ^**^P<0.01; ^***^P<0.001 compared with the control. Scale bars: 200 μm.

### Overexpression of miR-10b enhances osteogenesis, but suppresses adipogenesis of hADSCs

To investigate the biological function of miR-10b on osteogenic and adipogenic differentiation, we expressed the precursor of miR-10b with a lentiviral vector (lenti-10b) in hADSCs. The same lentiviral vector expressing a scrambled sequence with no homology to the human genome was used as a parallel control (lenti-NC). hADSCs were incubated with lentivirus for 24 h and amplified for 2 more days. GFP- positive cells were then purified by puromycin. MiRNA specific stem-loop RT-PCR confirmed that the expression level of miR-10b was dramatically upregulated by more than 500-fold in lenti-10b-infected hADSCs (Fig. 2A). Lenti-10b-infected hADSCs were induced to differentiate into the osteoblast lineage with osteogenic induction media. Alkaline phosphatase (ALP) staining and ALP activity assay showed that the expression and activity of ALP, an early marker of osteoblasts, was increased by miR-10b overexpression (Fig. 2B, C). Alizarin red staining indicated that matrix mineralization was enhanced in miR-10b-infected cells (Fig. 2D). qRT-PCR analysis showed that lenti-10b-infected hADSCs exhibited higher levels of ALP, OPN, RUNX2, OC, COL1A1 and IBSP than control cells (Fig. 2E). Western blot analysis showed that the protein levels of these markers were also increased in lenti-10b-infected hADSCs (Fig. 2F). These data demonstrated that overexpression of miR-10b promoted osteogenic differentiation of hADSCs *in vitro*. To further elucidate the effect of miR-10b on adipogenic differentiation, lenti-10b-infected hADSCs were induced toward the adipogenic lineage. Oil red O staining and extraction showed that overexpression of miR-10b resulted in a significant decrease in oil red O-positive adipocyte number (Fig. 2G, H). The expression of adipogenic transcription factors (C/EBPα, C/EBPβ and PPARγ) and marker genes (LPL and AP2) decreased at the mRNA and protein levels (Fig. 2I, J), indicating that upregulation of miR-10b can impair adipogenesis of hADSCs.

**Figure 5.**
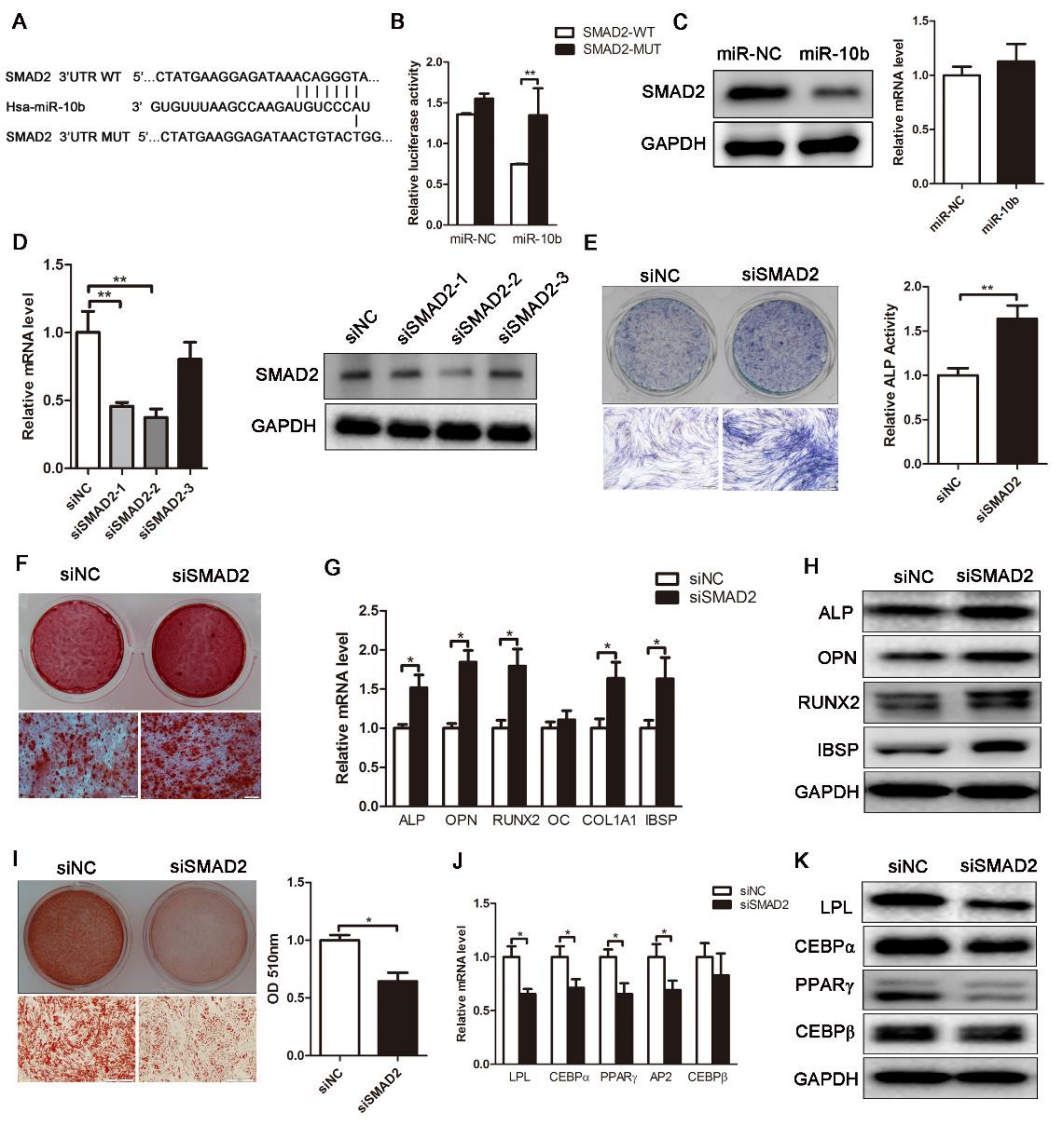
SMAD2 is a direct target of miR-10b. (A and B) Bioinformatic analysis was performed to predict the binding seed sequence of miR-10b with the 3’UTR of SMAD2. The wild-type (WT) or mutant-type (MUT) constructs were inserted into the psiCHECK-2 reporter vector. Luciferase activity was measured in the lysates, and the values were normalized to the psiCHECK-2 vector. (C) Western blot and qRT-PCR analyzed the expression of SMAD2 in hADSCs. (D) The knockdown efficiency of three SMAD2 siRNAs was confirmed by qRT-PCR and western blot. (E) ALP staining and activity assay were performed to analyze ALP expression and activity on day 6. (F) Alizarin red staining showed increased calcification after SMAD2 knockdown. (G and H) The critical regulators of osteogenesis were analyzed by qRT-PCR and western blot. (I) Oil red O staining and extraction were performed to detect the formation of lipid droplets on day 10 of adipogenic differentiation. (J and K) qRT-PCR and western blot were used to analyze adipogenic-specific markers after SMAD2 knockdown. The data, normalized to GAPDH, are averages of 3 independent experiments (mean ± SD). ^*^P<0.05; ^**^P<0.01 compared with the control. Scale bars: 200 μm.

### Overexpression of miR-10b enhances ectopic bone formation of hADSCs in vivo

Next, we explored the effect of miR-10b on bone formation *in vivo *by building a preclinical ectopic bone formation model in NOD/SCID mice. Lenti-NC or lenti-10b-infected hADSCs were loaded onto hydroxyapatite/tricalcium phosphate (HA/TCP) scaffolds, respectively, and implanted subcutaneously into NOD/SCID mice. The grafts were removed after 8 weeks, H&E staining, masson trichrome staining, safranin O staining and immunohistochemistry staining were used to evaluate ectopic bone formation. H&E staining revealed a remarkable increase in the amount of osteoid in the mouse grafts with lenti-10b-infected hADSCs, whereas less osteoid was detected in the grafts with either lenti-NC-infected hADSCs or uninfected hADSCs (Fig. 3A). Masson trichrome and safranin O staining revealed that collagen and cartilage were more abundant in miR-10b overexpressing hADSC xenografts than in the lenti-NC group (Fig. 3B, C). Immunohistochemical staining showed that the expression of ALP, OPN, RUNX2 and IBSP increased in the mouse grafts with lenti-10b-infected hADSCs compared with the other two groups (Fig. 3D). These results demonstrated that overexpression of miR-10b in hADSCs not only enhances osteogenic differentiation *in vitro* but also facilitates ectopic bone formation *in vivo*.

**Figure 6.**
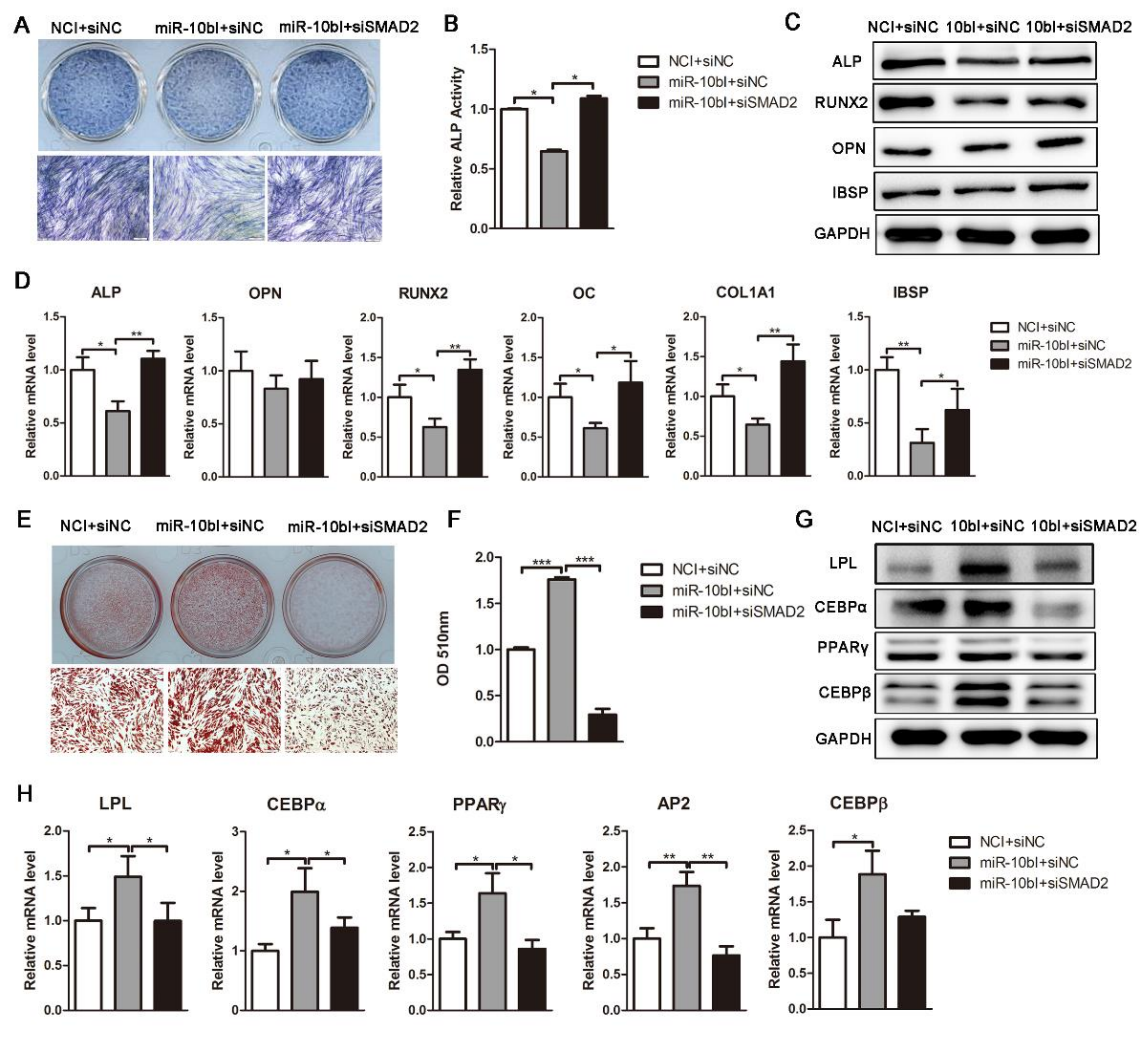
SMAD2 knockdown reverses the effect of miR-10bI on osteogenic and adipogenic differentiation of hADSCs. (A and B) ALP staining and activity were performed on day 6 of osteogenic differentiation. (C and D) Western blot and qRT-PCR were used to analyze osteogenic factor expression after different treatments. (E and F) Oil red O staining and extraction were performed to detect the lipid droplets formation on day 10 of adipogenic differentiation. (G and H) Western blot and qRT-PCR were performed to analyze protein and mRNA expression levels of adipogenic markers, respectively. The data, normalized to GAPDH, are averages of 3 independent experiments (mean ± SD). ^*^P<0.05; ^**^P<0.01 compared with the control. Scale bars: 200 μm.

### Downregulation of endogenous miR-10b suppresses osteogenesis, but promotes adipogenesis of hADSCs

To further confirm the effect of miR-10b on the differentiation of hADSCs, we next used specific inhibitors to suppress endogenous expression of miR-10b in hADSCs. Stem-loop RT-PCR analysis confirmed that transfection of miR-10b specific inhibitor (miR-10bI) effectively inhibited miR-10b expression in hADSCs (Fig. 4A). Then, miR-10b-downregulated cells were induced to differentiate into the osteogenic lineage. MiR-10bI transfection resulted in significantly decreased ALP staining and activity, reduced matrix mineralization, and suppressed expression of osteogenic transcription factors and markers at mRNA and protein levels (Fig. 4B-F), indicating that downregulation of miR-10b can inhibit osteogenesis of hADSCs. In contrast, enhanced oil red O staining and extraction, elevated expression of adipogenic-specific factors and marker genes at the mRNA and protein levels indicated that adipogenesis was dramatically promoted in miR-10b-inhibited cells (Fig. 4G-J). Hence, these data confirmed that inhibition of miR-10b significantly suppresses osteogenic differentiation but promotes adipogenic differentiation of hADSCs.

**Figure 7.**
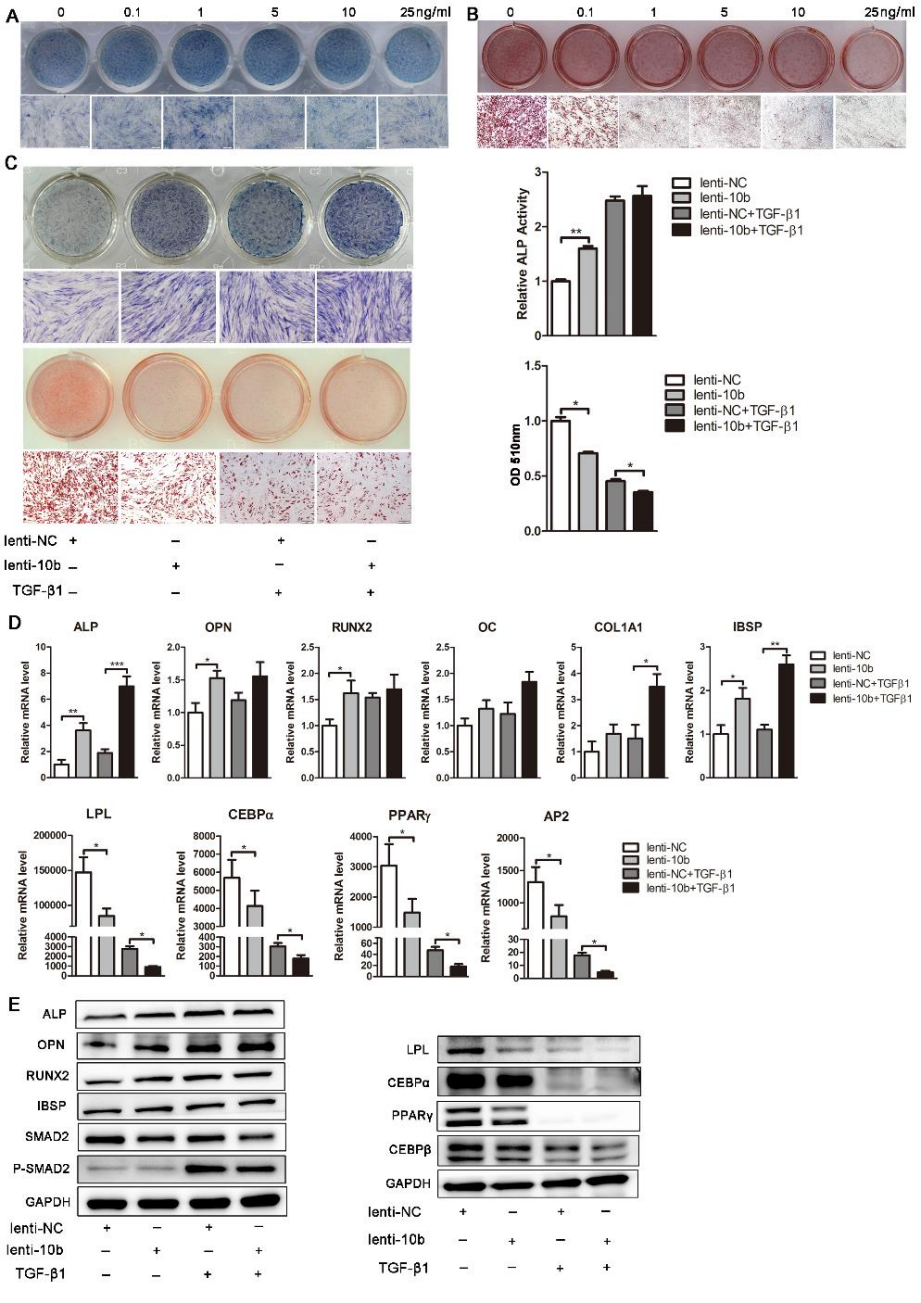
MiR-10b regulates hADSC differentiation partly through the TGF-β signaling pathway. (A) ALP staining was used to analyze the effects of different concentrations of TGF-β1 on ALP expression. (B) Oil red O staining was performed to analyze the effects of different concentrations of TGF-β1 on the formation of lipid droplets. (C) ALP staining and activity were detected on day 6 of osteogenic differentiation. Oil red O staining and extraction were analyzed on day 10 of adipogenic differentiation after different treatments. (D) The mRNA levels of osteogenic- and adipogenic-related genes after different treatments. (E) Western blot analyzed the protein levels of the osteogenic and adipogenic factors, and TGF-β signaling pathway-related molecular elements after different treatments. The data, normalized to GAPDH are averages of 3 independent experiments (mean±SD). ^*^P<0.05; ^**^P<0.01; ^***^P<0.001 compared with the control. Scale bars: 200 μm.

### MiR-10b directly targets SMAD2

To reveal the molecular mechanism by which miR-10b regulates osteogenic and adipogenic differentiation of hADSCs, TargetScan was used to predict potential targets of miR-10b. Among the candidates, we found that osteoblast or adipocyte-related genes NFAT5, ESRRG, SMAD2, SMURF1 and HDAC4 have miR-10b binding sites in their 3’UTR (Fig. 5A; Supplementary Fig. 2). To investigate whether miR-10b directly targets these genes, we constructed luciferase reporters that had either a wild-type (WT) 3’UTR or a 3’UTR-containing mutant (MUT) sequence of the miR-10b binding site. Dual luciferase reporter analysis showed that overexpression of miR-10b significantly inhibited the luciferase reporter activity of the vector containing WT SMAD2 3’UTR, but not other genes (Fig. 5B; Supplementary Fig. 2). Moreover, the expression of SMAD2 in miR-10b-overexpressed hADSCs was markedly downregulated at the protein level but not at the mRNA level (Fig. 5C), suggesting that miR-10b regulates SMAD2 expression at the post-transcriptional level in hADSCs. To investigate whether miR-10b functionally targets SMAD2 in regulating hADSC osteogenic and adipogenic differentiation, we suppressed expression of SMAD2 by transfecting hADSCs with siRNAs against SMAD2. The data showed that SMAD2 was significantly suppressed by siSMAD2-2 at both mRNA and protein levels (Fig. 5D). Therefore, siSMAD2-2 was chosen for further use in subsequent functional experiments. The data showed that SMAD2 downregulation increased osteogenic differentiation of hADSCs, as demonstrated by increased ALP staining and ALP activity (Fig. 5E), enhanced mineral deposition (Fig. 5F), and upregulated ALP, OPN, RUNX2, COL1A1 and IBSP at the mRNA and protein levels (Fig. 5G, H). These results were consistent with those observed in miR-10b-overexpressed hADSCs. To investigate the effect of SMAD2 on adipogenic differentiation, SMAD2-downregulated hADSCs were differentiated into adipocytes. The data revealed that inhibited endogenous expression of SMAD2 remarkably suppressed adipogenic differentiation of hADSCs, as indicated by weakened oil red O staining and decreased OD510 nm value of the oil red O extraction (Fig. 5I), as well as reduced expression of the adipogenic regulators and adipocyte markers at the mRNA and protein levels (Fig. 5J, K). Taken together, these results revealed that SMAD2 is a potential target of miR-10b in regulating hADSC differentiation.

**Figure 8.**
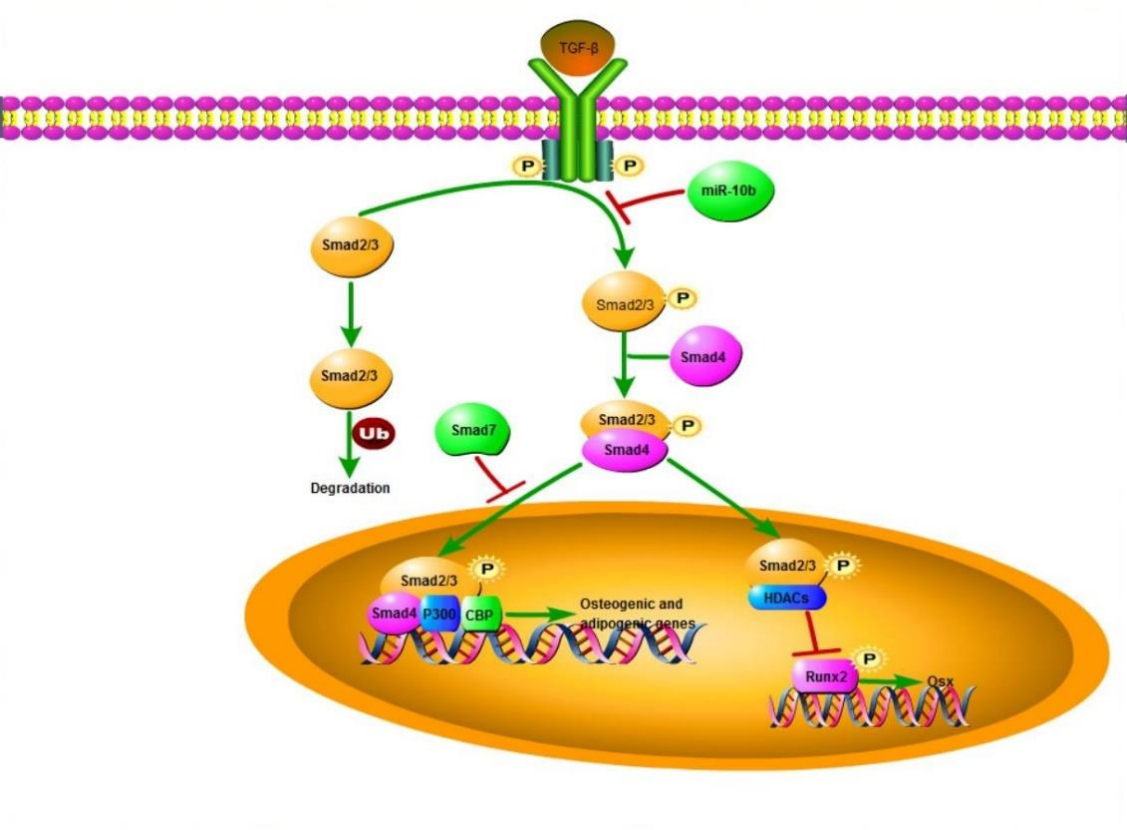
A schematic model illustrating that miR-10b mediated function in osteogenic and adipogenic differentiation of hADSCs. miR-10b suppresses SMAD2 expression at the post-transcriptional level, resulting in downregulation of the TGF-β signaling pathway, and thereby promoting osteogenic differentiation and decreasing adipogenic differentiation.

### Knockdown of SMAD2 can remedy the effect of endogenous miR-10b reduction on osteogenesis and adipogenesis

To further confirm that the effect of miR-10b during osteogenic and adipogenic differentiation is mediated by targeting SMAD2, we silenced miR-10b and then knockdown SMAD2 in hADSCs. The results showed that SMAD2 knockdown effectively abolished the suppression of miR-10bI on osteogenic differentiation, as demonstrated by enhanced ALP staining and activity and increased expression of osteoblast-specific factors (Fig. 6A-D). SMAD2 knockdown also effectively reversed the promotion effect of miR-10bI on hADSC adipogenic differentiation, as indicated by reduced oil red O staining and extraction, and decreased expression of adipogenic markers (Fig. 6E-H). These results demonstrated that deletion of its target could block the effect of miR-10bI, further indicating that miR-10b regulates the osteogenesis and adipogenesis of hADSCs through direct targeting SMAD2.

### MiR-10b regulates lineage commitment of hADSCs partly via TGF-β signaling pathway

SMAD2 is identified as an important receptor of the TGF-β signaling pathway. To verify whether miR-10b regulates the balance of osteogenic and adipogenic differentiation through the TGF-β signaling pathway, recombinant human TGF-β1 was added to the osteogenic and adipogenic induction medium during hADSC differentiation. Consistent with previous research [38-40], we found that low concentrations of TGF-β1 (0.1-1 ng/ml) stimulated osteogenic differentiation, while high concentrations (5-25 ng/ml) inhibited osteogenesis (Fig. 7A; Supplementary Fig. 3). In addition, TGF-β1 has a strong inhibitory effect on adipogenesis of hADSCs, as indicated by the dramatically reduced numbers of oil red O-positive adipocytes and the decreased expression of adipogenic marker genes (Fig. 7B; Supplementary Fig. 3). Furthermore, when 1 ng/ml TGF-β1 was added to the osteogenic induction medium, the enhanced osteogenic differentiation by miR-10b overexpression was further promoted by TGF-β1, as demonstrated by enhanced ALP staining, increased ALP activity and upregulated osteogenic factor expression (Fig. 7C-E). In addition, the enhancement of hADSC osteogenic differentiation by miR-10b overexpression was almost blocked by high concentration of TGF-β1 (5 ng/ml), as demonstrated by decreased ALP staining, ALP activity and reduced mRNA expression of osteogenic factors (Supplementary Fig. 4). However, the inhibitory effects of miR-10b on adipogenesis were not significantly attenuated by TGF-β1, as indicated by oil red O staining and extraction, and the expression of adipogenic markers at the mRNA and protein levels (Fig. 7C-E). Together, these results demonstrated that miR-10b regulates osteogenic and adipogenic differentiation of hADSCs partly via suppressing the TGF-β signaling pathway (Fig. 8).

## DISCUSSION

A growing number of miRNAs have been found to be critical factors involved in the regulation of osteogenic or adipogenic differentiation. However, only a few miRNAs have been identified to be responsible for maintaining the balance of adipogenesis and osteogenesis. In this study, we first demonstrated that miR-10b expression was inversely changed during the osteogenic and adipogenic differentiation of hADSCs. In the clinical osteoporosis samples, miR-10b was positively correlated with bone formation markers and negatively correlated with adipocyte formation genes. Functional and mechanistic analyses revealed that miR-10b can effectively promote osteogenic differentiation, suppress adipogenic differentiation *in vitro*, and enhance bone tissue formation *in vivo*, via suppressing the TGF-β/SMAD2 signaling pathway. Our findings suggested that miR-10b may represent as a potential therapeutic target and biomarker for treating bone osteogenic-adipogenic metabolic disorders.

Both osteogenesis and adipogenesis of MSCs are important physiological processes in controlling body homeostasis. Once the balance between adipocyte and osteoblast differentiation of MSCs is disrupted, various metabolic disorders will occur [41, 42]. Clinically, in osteoporosis, the most common bone remodeling disorder worldwide, adipocyte tissue volume in bone marrow is increased [4, 43]. Accumulating evidence has demonstrated that multiple regulatory factors and signaling pathways are involved in regulating the lineage commitment of MSCs, including TGF-β/BMP, Wnt/β-catenin, Hedgehog, Notch, JAK/STAT, MAPK and PI3K/AKT [44-46]. These signaling pathways do not function in isolation, and the lineage commitment of MSCs is mediated by a complex network of various signaling pathways. Therefore, it is urgent to elucidate what factors are involved and how they orchestrate to regulate the specification of cell fate.

miRNAs, which were previously considered “junk” RNA, useless for the individual, are now regarded as critical regulators for most cellular events. By targeting hundreds of mRNAs, miRNAs could switch cell fate and fine tune genome expression. miR-637 has been reported to promote adipogenesis and suppress osteogenesis of hMSCs through direct suppression of Osterix expression [47]. Our previous study found that miR-17-5p and miR-106a can suppress osteoblast differentiation and promote adipocyte differentiation of hADSCs by direct repression of the BMP2/SMAD5 pathway [25]. In this study, we revealed that miR-10b can significantly enhance osteogenic differentiation and inhibit adipogenic differentiation of hADSCs by directly suppressing SMAD2.

Both SMAD5 and SMAD2 belong to the TGF-β superfamily. SMAD proteins act as critical intracellular receptors and participate in canonical TGF-β and BMP superfamily pathways. Activated receptor-regulated SMADs (R-SMADs) include SMAD 1, 2, 3, 5 and 8 [48]. SMAD2/3 are activated by TGF-β receptors and mediate TGF-β responses, whereas SMAD1/5/8 are activated by BMP receptors and transduce BMP signals [49]. SMAD acts as regulator of osteogenesis and bone regeneration in a series of mechanisms, such as in interactions with RUNX2, the dominant regulator of osteogenesis and a modulator of the balance between adipogenesis and osteogenesis [50]. It has been reported that SMAD2, SMAD3 and SMAD5 physically interact with RUNX2 [51]. SMAD2/3 inhibit RUNX2 expression, and activated SMAD3 also recruits class II histone deacetylases (HDACs) 4 and 5 to repress the function of RUNX2 [52, 53], whereas SMAD5 promotes RUNX2 expression [54]. Stable overexpression of SMAD2 or SMAD3 inhibited adipogenic differentiation in the preadipocyte cell line 3T3-F442A [55]. These studies implied that distinct relationships may exist between each SMAD protein and RUNX2. In this study, we found that downregulation of SMAD2 can upregulate RUNX2 expression, promote osteoblast differentiation and suppress adipocyte differentiation and knockdown of SMAD2 can rescue the effect of endogenous miR-10b reduction on osteogenesis and adipogenesis. We demonstrated that miR-10b indirectly regulates RUNX2 by directly suppressing the expression of SMAD2. Enhanced RUNX2 activity by suppressed SMAD2 level might be partially responsible for the effect of miR-10b on the balance of osteogenesis and adipogenesis.

TGF-β acts as a central coordinator in maintaining postnatal bone mass by coupling bone resorption and bone formation [56, 57]. There are three TGF-βs in mammals: TGF-β1, TGF-β2 and TGF-β3 [58]. TGF-β1 is secreted by osteoblasts and bone marrow MSCs and is stored in bone matrix. TGF-β1 acts as a double-edged sword in the maintenance of bone remodeling, and the effect of TGF-β1 on *in vitro* osteogenic differentiation of MSCs is highly dependent on the specific culture conditions such as cell density, the dosage and the presence of serum. Low concentrations of TGF-β1 (0.1-1 ng/ml) stimulate osteoblast differentiation, while high concentrations of TGF-β1 (10 ng/ml) inhibit this process. TGF-β1 inhibits adipogenic differentiation of MSCs in monolayer culture [59]. It was reported that the regulation of TGF-β1 in MSC osteogenic differentiation and adipogenic differentiation occurs through activating TAZ by the SMAD-dependent pathway [40]. Therefore, fine tuning TGF-β1 levels is significant for the coupling of bone formation [60]. In the present study, to confirm activation of the intracellular TGF-β signaling pathway, we evaluated the phosphorylated status of SMAD2. We demonstrated a greater amount of phosphorylated SMAD2 (p-SMAD2) after TGF-β1 was added, however, the total SMAD2 level only mildly decreased. Consistent with previous research [38-40], we found that low concentrations of TGF-β1 (0.1-1 ng/ml) can stimulate osteogenic differentiation while high concentrations (5-25 ng/ml) inhibit osteogenesis. However, both high and low concentrations of TGF-β1 have a strong inhibitory effect on adipogenesis of hADSCs. Moreover, the enhancement of osteogenic differentiation by miR-10b overexpression was almost blocked by high concentration of TGF-β1, while the inhibitory effects of miR-10b on adipogenesis were not significantly attenuated by TGF-β1. TGF-β1 can mimic the deficiency effect of miR-10b on osteogenesis, however, it cannot simulate the function of miR-10b on adipogenesis, suggesting that miR-10b regulates the balance of osteogenic and adipogenic differentiation by directly targeting SMAD2 partly through the TGF-β pathway. Other signaling pathways might also participate in this process.

In conclusion, we first demonstrated that miR-10b, which is a well-studied miRNA in various types of cancers, was positively correlated with bone formation and negatively correlated with adipocyte formation in clinical osteoporosis samples, promoted osteogenesis and suppressed adipogenesis *in vitro* and enhanced ectopic bone formation *in vivo*. miR-10b regulated osteogenic and adipogenic lineage commitment of hADSCs by directly targeting SMAD2 and subsequently increased osteogenic gene expression and decreased adipogenic gene expression. By targeting SMAD2, miR-10b enhanced osteogenesis via the TGF-β signaling pathway, while it impaired adipogenesis primarily through other pathways. Our study illustrates a potential crucial function of miR-10b in the development of osteoporosis, and miR-10b may be a promising therapeutic agent for treating osteoporosis or other bone-fat metabolism diseases.

## Supplementary Data

Supplementary Data are avalible online at www.aginganddisease.org/EN/10.14336/AD.2018.0214

## References

[1] Dall TM, Gallo PD, Chakrabarti R, West T, Semilla AP, Storm MV (2013). An aging population and growing disease burden will require a large and specialized health care workforce by 2025. Health Aff (Millwood), 32: 2013–20. 24191094 10.1377/hlthaff.2013.0714

[2] Gibon E, Lu LY, Nathan K, Goodman SB (2017). Inflammation, ageing, and bone regeneration. J Orthop Translat, 10: 28–35. 29094003 10.1016/j.jot.2017.04.002 PMC5662134

[3] MacLean C, Newberry S, Maglione M, McMahon M, Ranganath V, Suttorp M, et al (2008). Systematic review: comparative effectiveness of treatments to prevent fractures in men and women with low bone density or osteoporosis. Ann Intern Med, 148: 197–213. 18087050 10.7326/0003-4819-148-3-200802050-00198

[4] Meunier P, Aaron J, Edouard C, Vignon G (1971). Osteoporosis and the replacement of cell populations of the marrow by adipose tissue. A quantitative study of 84 iliac bone biopsies. Clin Orthop Relat Res, 80: 147–54. 5133320 10.1097/00003086-197110000-00021

[5] Chamberlain G, Fox J, Ashton B, Middleton J (2007). Concise review: mesenchymal stem cells: their phenotype, differentiation capacity, immunological features, and potential for homing. Stem Cells, 25: 2739–49. 17656645 10.1634/stemcells.2007-0197

[6] Bennett JH, Joyner CJ, Triffitt JT, Owen ME (1991). Adipocytic cells cultured from marrow have osteogenic potential. J Cell Sci, 99(Pt 1): 131–9. 1757497 10.1242/jcs.99.1.131

[7] Beresford JN, Bennett JH, Devlin C, Leboy PS, Owen ME (1992). Evidence for an inverse relationship between the differentiation of adipocytic and osteogenic cells in rat marrow stromal cell cultures. J Cell Sci, 102(Pt 2): 341–51. 1400636 10.1242/jcs.102.2.341

[8] Dorheim MA, Sullivan M, Dandapani V, Wu X, Hudson J, Segarini PR, et al (1993). Osteoblastic gene expression during adipogenesis in hematopoietic supporting murine bone marrow stromal cells. J Cell Physiol, 154: 317–28. 8425912 10.1002/jcp.1041540215

[9] Houghton A, Oyajobi BO, Foster GA, Russell RG, Stringer BM (1998). Immortalization of human marrow stromal cells by retroviral transduction with a temperature sensitive oncogene: identification of bipotential precursor cells capable of directed differentiation to either an osteoblast or adipocyte phenotype. Bone, 22: 7–16. 9437508 10.1016/s8756-3282(97)00229-9

[10] Levi B, Longaker MT (2011). Concise review: adipose-derived stromal cells for skeletal regenerative medicine. Stem Cells, 29: 576–82. 21305671 10.1002/stem.612 PMC3323288

[11] Shukla GC, Singh J, Barik S (2011). MicroRNAs: Processing, Maturation, Target Recognition and Regulatory Functions. Mol Cell Pharmacol, 3: 83–92. 22468167 PMC3315687

[12] Wienholds E, Koudijs MJ, van Eeden FJ, Cuppen E, Plasterk RH (2003). The microRNA-producing enzyme Dicer1 is essential for zebrafish development. Nat Genet, 35: 217–8. 14528306 10.1038/ng1251

[13] Dickins RA, Hemann MT, Zilfou JT, Simpson DR, Ibarra I, Hannon GJ, et al (2005). Probing tumor phenotypes using stable and regulated synthetic microRNA precursors. Nat Genet, 37: 1289–95. 16200064 10.1038/ng1651

[14] Cimmino A, Calin GA, Fabbri M, Iorio MV, Ferracin M, Shimizu M, et al (2005). miR-15 and miR-16 induce apoptosis by targeting BCL2. Proc Natl Acad Sci U S A, 102: 13944–9. 16166262 10.1073/pnas.0506654102 PMC1236577

[15] Chen CZ, Li L, Lodish HF, Bartel DP (2004). MicroRNAs modulate hematopoietic lineage differentiation. Science, 303: 83–6. 14657504 10.1126/science.1091903

[16] Kim YJ, Bae SW, Yu SS, Bae YC, Jung JS (2009). miR-196a regulates proliferation and osteogenic differentiation in mesenchymal stem cells derived from human adipose tissue. J Bone Miner Res, 24: 816–25. 19063684 10.1359/jbmr.081230

[17] Zhang J, Tu Q, Bonewald LF, He X, Stein G, Lian J, et al (2011). Effects of miR-335-5p in modulating osteogenic differentiation by specifically downregulating Wnt antagonist DKK1. J Bone Miner Res, 26: 1953–63. 21351149 10.1002/jbmr.377 PMC3810406

[18] Li H, Li T, Fan J, Li T, Fan L, Wang S, et al (2015). miR-216a rescues dexamethasone suppression of osteogenesis, promotes osteoblast differentiation and enhances bone formation, by regulating c-Cbl-mediated PI3K/AKT pathway. Cell Death Differ, 22: 1935–45. 26206089 10.1038/cdd.2015.99 PMC4816120

[19] Luzi E, Marini F, Sala SC, Tognarini I, Galli G, Brandi ML (2008). Osteogenic differentiation of human adipose tissue-derived stem cells is modulated by the miR-26a targeting of the SMAD1 transcription factor. J Bone Miner Res, 23: 287–95. 18197755 10.1359/jbmr.071011

[20] Li Z, Hassan MQ, Volinia S, van Wijnen AJ, Stein JL, Croce CM, et al (2008). A microRNA signature for a BMP2-induced osteoblast lineage commitment program. Proc Natl Acad Sci U S A, 105: 13906–11. 18784367 10.1073/pnas.0804438105 PMC2544552

[21] Esau C, Kang X, Peralta E, Hanson E, Marcusson EG, Ravichandran LV, et al (2004). MicroRNA-143 regulates adipocyte differentiation. J Biol Chem, 279: 52361–5. 15504739 10.1074/jbc.C400438200

[22] Chen Y, Siegel F, Kipschull S, Haas B, Fröhlich H, Meister G, et al (2013). miR-155 regulates differentiation of brown and beige adipocytes via a bistable circuit. Nat Commun, 4: 1769. 23612310 10.1038/ncomms2742 PMC3644088

[23] Zaragosi LE, Wdziekonski B, Brigand KL, Villageois P, Mari B, Waldmann R, et al (2011). Small RNA sequencing reveals miR-642a-3p as a novel adipocyte-specific microRNA and miR-30 as a key regulator of human adipogenesis. Genome Biol, 12: R64. 21767385 10.1186/gb-2011-12-7-r64 PMC3218826

[24] Huang J, Zhao L, Xing L, Chen D (2010). MicroRNA-204 regulates Runx2 protein expression and mesenchymal progenitor cell differentiation. Stem Cells, 28: 357–64. 20039258 10.1002/stem.288 PMC2837600

[25] Li H, Li T, Wang S, Wei J, Fan J, Li J, et al (2013). miR-17-5p and miR-106a are involved in the balance between osteogenic and adipogenic differentiation of adipose-derived mesenchymal stem cells. Stem Cell Res, 10: 313–24. 23399447 10.1016/j.scr.2012.11.007

[26] Wang J, Guan X, Guo F, Zhou J, Chang A, Sun B, et al (2013). miR-30e reciprocally regulates the differentiation of adipocytes and osteoblasts by directly targeting low-density lipoprotein receptor-related protein 6. Cell Death Dis, 4: e845. 24113179 10.1038/cddis.2013.356 PMC3824666

[27] Jeong BC, Kang IH, Hwang YC, Kim SH, Koh JT (2014). MicroRNA-194 reciprocally stimulates osteogenesis and inhibits adipogenesis via regulating COUP-TFII expression. Cell Death Dis, 5: e1532. 25412310 10.1038/cddis.2014.485 PMC4260743

[28] Ma L, Teruya-Feldstein J, Weinberg RA (2007). Tumour invasion and metastasis initiated by microRNA-10b in breast cancer. Nature, 449: 682–8. 17898713 10.1038/nature06174

[29] Saldanha G, Elshaw S, Sachs P, Alharbi H, Shah P, Jothi A, et al (2016). microRNA-10b is a prognostic biomarker for melanoma. Mod Pathol, 29: 112–21. 26743475 10.1038/modpathol.2015.149

[30] Nakata K, Ohuchida K, Mizumoto K, Kayashima T, Ikenaga N, Sakai H, et al (2011). MicroRNA-10b is overexpressed in pancreatic cancer, promotes its invasiveness, and correlates with a poor prognosis. Surgery, 150: 916–22. 22018284 10.1016/j.surg.2011.06.017

[31] Li QJ, Zhou L, Yang F, Wang GX, Zheng H, Wang DS, et al (2012). MicroRNA-10b promotes migration and invasion through CADM1 in human hepatocellular carcinoma cells. Tumour Biol, 33: 1455–65. 22528944 10.1007/s13277-012-0396-1

[32] Nishida N, Yamashita S, Mimori K, Sudo T, Tanaka F, Shibata K, et al (2012). MicroRNA-10b is a prognostic indicator in colorectal cancer and confers resistance to the chemotherapeutic agent 5-fluorouracil in colorectal cancer cells. Ann Surg Oncol, 19: 3065–71. 22322955 10.1245/s10434-012-2246-1

[33] Liu Y, Li M, Zhang G, Pang Z (2013). MicroRNA-10b overexpression promotes non-small cell lung cancer cell proliferation and invasion. Eur J Med Res, 18: 41. 24216130 10.1186/2047-783X-18-41 PMC4177004

[34] Nakayama I, Shibazaki M, Yashima-Abo A, Miura F, Sugiyama T, Masuda T, et al (2013). Loss of HOXD10 expression induced by upregulation of miR-10b accelerates the migration and invasion activities of ovarian cancer cells. Int J Oncol, 43: 63–71. 23670532 10.3892/ijo.2013.1935

[35] Cao Y, Sun Z, Liao L, Meng Y, Han Q, Zhao RC (2005). Human adipose tissue-derived stem cells differentiate into endothelial cells in vitro and improve postnatal neovascularization in vivo. Biochem Biophys Res Commun, 332: 370–9. 15896706 10.1016/j.bbrc.2005.04.135

[36] Fan H, Qiao L, Kang KD, Fan J, Wei W, Luo G (2017). Attachment and Postattachment Receptors Important for Hepatitis C Virus Infection and Cell-to-Cell Transmission. J Virol, 91. 10.1128/JVI.00280-17 PMC5469255 28404852

[37] Abdallah BM, Ditzel N, Kassem M (2008). Assessment of bone formation capacity using in vivo transplantation assays: procedure and tissue analysis. Methods Mol Biol, 455: 89–100. 18463812 10.1007/978-1-59745-104-8_6

[38] Lieb E, Vogel T, Milz S, Dauner M, Schulz MB (2004). Effects of transforming growth factor beta1 on bonelike tissue formation in three-dimensional cell culture. II: Osteoblastic differentiation. Tissue Eng, 10: 1414–25. 15588401 10.1089/ten.2004.10.1414

[39] Liu P, Oyajobi BO, Russell RG, Scutt A (1999). Regulation of osteogenic differentiation of human bone marrow stromal cells: interaction between transforming growth factor-beta and 1,25(OH)(2) vitamin D(3) In vitro. Calcif Tissue Int, 65: 173–80. 10430653 10.1007/s002239900678

[40] Zhao L, Jiang S, Hantash BM (2010). Transforming growth factor beta1 induces osteogenic differentiation of murine bone marrow stromal cells. Tissue Eng Part A, 16: 725–33. 19769530 10.1089/ten.TEA.2009.0495

[41] Teitelbaum SL (2000). Bone resorption by osteoclasts. Science, 289: 1504–8. 10968780 10.1126/science.289.5484.1504

[42] Delany AM, Amling M, Priemel M, Howe C, Baron R, Canalis E (2000). Osteopenia and decreased bone formation in osteonectin-deficient mice. J Clin Invest, 105: 915–23. 10749571 10.1172/JCI7039 PMC377474

[43] Justesen J, Stenderup K, Ebbesen EN, Mosekilde L, Steiniche T, Kassem M (2001). Adipocyte tissue volume in bone marrow is increased with aging and in patients with osteoporosis. Biogerontology, 2: 165–71. 11708718 10.1023/a:1011513223894

[44] Chen Q, Shou P, Zheng C, Jiang M, Cao G, Yang Q, et al (2016). Fate decision of mesenchymal stem cells: adipocytes or osteoblasts. Cell Death Differ, 23: 1128–39. 26868907 10.1038/cdd.2015.168 PMC4946886

[45] Rosen ED, MacDougald OA (2006). Adipocyte differentiation from the inside out. Nat Rev Mol Cell Biol, 7: 885–96. 17139329 10.1038/nrm2066

[46] Huang W, Yang S, Shao J, Li YP (2007). Signaling and transcriptional regulation in osteoblast commitment and differentiation. Front Biosci, 12: 3068–92. 17485283 10.2741/2296 PMC3571113

[47] Zhang JF, Fu WM, He ML, Wang H, Wang WM, Yu SC, et al (2011). MiR-637 maintains the balance between adipocytes and osteoblasts by directly targeting Osterix. Mol Biol Cell, 22: 3955–61. 21880893 10.1091/mbc.E11-04-0356 PMC3204058

[48] Song B, Estrada KD, Lyons KM (2009). Smad signaling in skeletal development and regeneration. Cytokine Growth Factor Rev, 20: 379–88. 19926329 10.1016/j.cytogfr.2009.10.010 PMC2825570

[49] Heldin CH, Miyazono K, ten DP (1997). TGF-beta signalling from cell membrane to nucleus through SMAD proteins. Nature, 390: 465–71. 9393997 10.1038/37284

[50] Afzal F, Pratap J, Ito K, Ito Y, Stein JL, van Wijnen AJ, et al (2005). Smad function and intranuclear targeting share a Runx2 motif required for osteogenic lineage induction and BMP2 responsive transcription. J Cell Physiol, 204: 63–72. 15573378 10.1002/jcp.20258

[51] Hanai J, Chen LF, Kanno T, Ohtani-Fujita N, Kim WY, Guo WH, et al (1999). Interaction and functional cooperation of PEBP2/CBF with Smads. Synergistic induction of the immunoglobulin germline Calpha promoter. J Biol Chem, 274: 31577–82. 10531362 10.1074/jbc.274.44.31577

[52] Kang JS, Alliston T, Delston R, Derynck R (2005). Repression of Runx2 function by TGF-beta through recruitment of class II histone deacetylases by Smad3. EMBO J, 24: 2543–55. 15990875 10.1038/sj.emboj.7600729 PMC1176457

[53] Li J, Tsuji K, Komori T, Miyazono K, Wrana JL, Ito Y, et al (1998). Smad2 overexpression enhances Smad4 gene expression and suppresses CBFA1 gene expression in osteoblastic osteosarcoma ROS17/2.8 cells and primary rat calvaria cells. J Biol Chem, 273: 31009–15. 9812998 10.1074/jbc.273.47.31009

[54] Lee KS, Kim HJ, Li QL, Chi XZ, Ueta C, Komori T, et al (2000). Runx2 is a common target of transforming growth factor beta1 and bone morphogenetic protein 2, and cooperation between Runx2 and Smad5 induces osteoblast-specific gene expression in the pluripotent mesenchymal precursor cell line C2C12. Mol Cell Biol, 20: 8783–92. 11073979 10.1128/mcb.20.23.8783-8792.2000 PMC86511

[55] Choy L, Skillington J, Derynck R (2000). Roles of autocrine TGF-beta receptor and Smad signaling in adipocyte differentiation. J Cell Biol, 149: 667–82. 10791980 10.1083/jcb.149.3.667 PMC2174852

[56] Tang Y, Wu X, Lei W, Pang L, Wan C, Shi Z, et al (2009). TGF-beta1-induced migration of bone mesenchymal stem cells couples bone resorption with formation. Nat Med, 15: 757–65. 19584867 10.1038/nm.1979 PMC2727637

[57] Wu M, Chen G, Li YP (2016). TGF-β and BMP signaling in osteoblast, skeletal development, and bone formation, homeostasis and disease. Bone Res, 4: 16009. 27563484 10.1038/boneres.2016.9 PMC4985055

[58] Feng XH, Derynck R (2005). Specificity and versatility in tgf-beta signaling through Smads. Annu Rev Cell Dev Biol, 21: 659–93. 16212511 10.1146/annurev.cellbio.21.022404.142018

[59] Choy L, Derynck R (2003). Transforming growth factor-beta inhibits adipocyte differentiation by Smad3 interacting with CCAAT/enhancer-binding protein (C/EBP) and repressing C/EBP transactivation function. J Biol Chem, 278: 9609–19. 12524424 10.1074/jbc.M212259200

[60] Janssens K, ten DP, Janssens S, Van Hul W (2005). Transforming growth factor-beta1 to the bone. Endocr Rev, 26: 743–74. 15901668 10.1210/er.2004-0001

