# Erratum to “MiRNA-10b Reciprocally Stimulates Osteogenesis and Inhibits Adipogenesis Partly through the TGF-β/SMAD2 Signaling Pathway”

**DOI:** 10.14336/AD.2021.0331

**Published:** 2021-10-01

**Authors:** Hongling Li, Junfen Fan, Linyuan Fan, Tangping Li, Yanlei Yang, Haoying Xu, Luchan Deng, Jing Li, Tao Li, Xisheng Weng, Shihua Wang, Robert Chunhua Zhao

We have noticed inadvertent errors in our article published in the December 2018 issue of Aging Dis (2018 9(6):1058-1073). In Figure 3A, the images for the HA/TCP (control) and HA/TCP+lenti-NC (negative control), and in Figure 3D, the image of HA/TCP-lenti-NC for IBSP have been presented incorrectly. The Figure 4G was incorret. We have attached corrected Figure 3 and Figure 4. The errors do not change the scientific conclusions of the article. The authors apologize for the errors.

**Figure 3.**
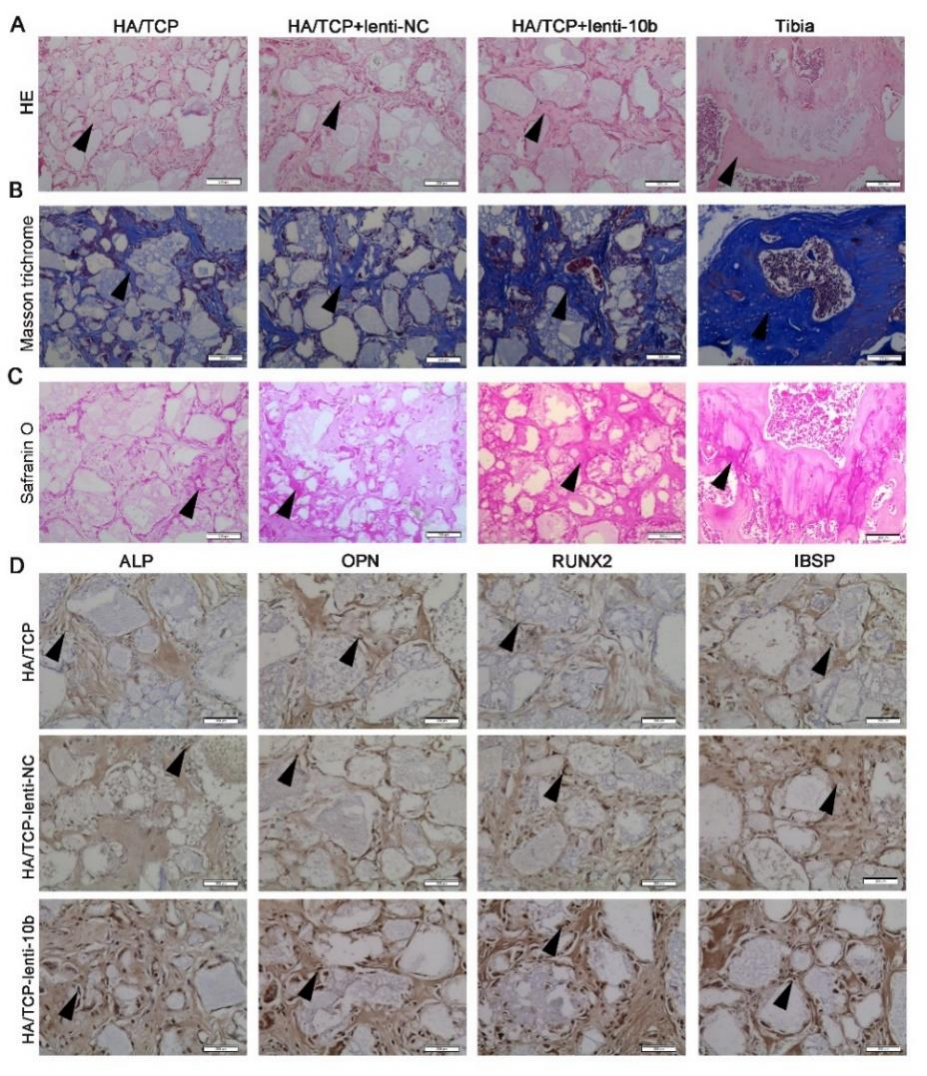
miR-10b promotes the ectopic bone formation of hADSCs *in vivo*. (A) H&E staining was used to analyze osteoid formation in the xenografts. (B) Masson trichrome staining indicated collagen. (C) Safranin O staining revealed cartilage formation in the xenografts. (D) Immunohistochemical staining showed the expression levels of osteogenic markers in the xenografts. Black arrows represent positive signals. Scale bars: 200 μm.

**Figure 4.**
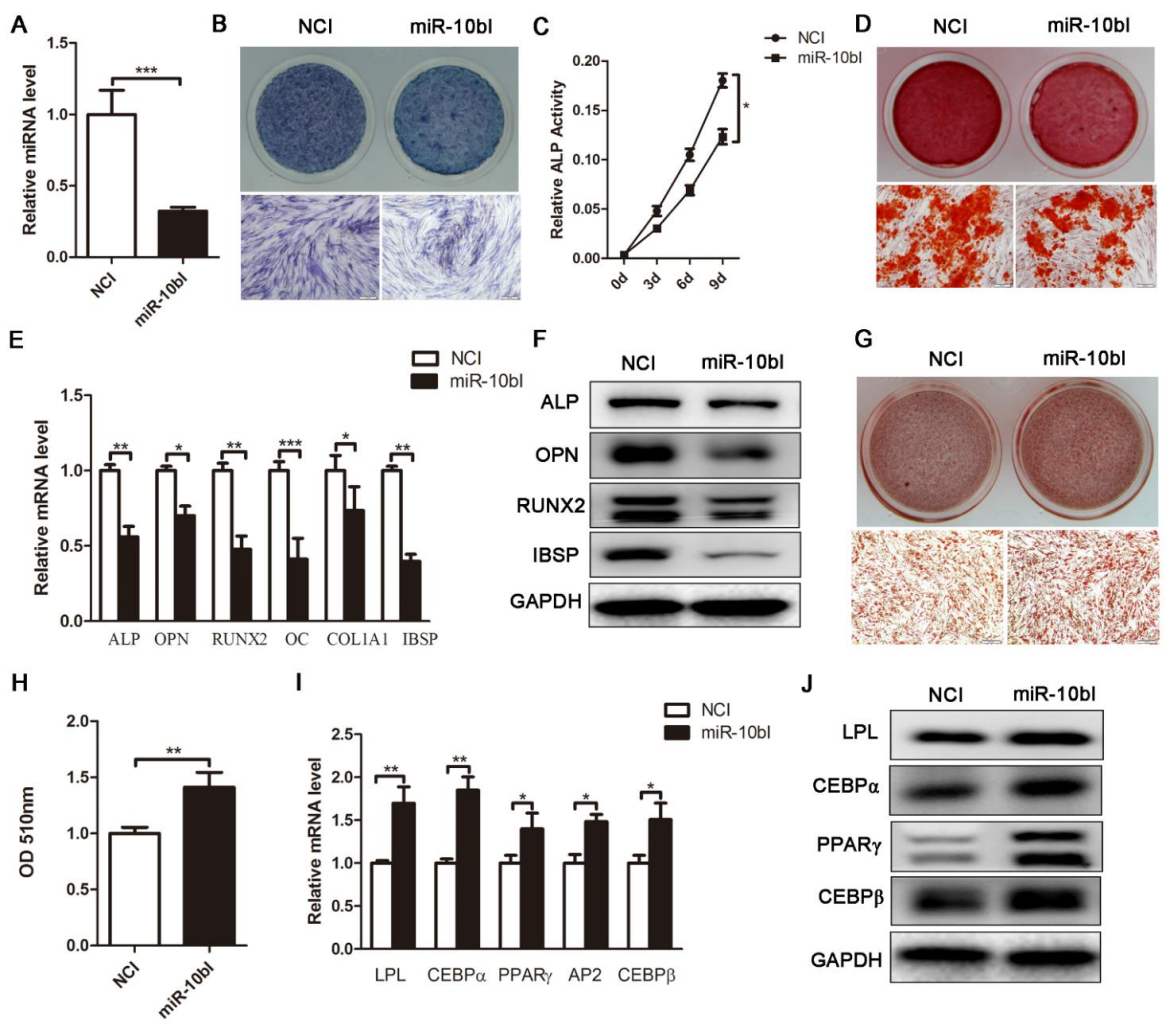
Downregulation of endogenous miR-10b suppresses osteogenic differentiation and enhances adipogenic differentiation. (A) miR-10b expression was determined by stem-loop RT-PCR. (B and C) ALP staining and ALP activity were performed after inhibiting miR-10b. (D) Alizarin red staining was performed on day 12. (E and F) qRT-PCR and western blot were performed to analyze the mRNA and protein levels of osteogenic-specific markers after miR-10b inhibitor transfection. (G and H) Oil red O staining and extraction were performed to detect the formation of lipid droplets on day 10 of adipogenic differentiation. (I and J) The expression of adipogenic-specific markers was analyzed by qRT-PCR and western blot. The data, normalized to GAPDH, are averages of 3 independent experiments (mean ± SD). ^*^P<0.05; ^**^P<0.01; ^***^P<0.001 compared with the control. Scale bars: 200 μm.

