# Supplementary material for: MiRNA-10b Reciprocally Stimulates Osteogenesis and Inhibits Adipogenesis Partly through the TGF-β/SMAD2 Signaling Pathway
Source: Aging Dis. 2018 Dec 4;9(6):1058–73. doi: 10.14336/AD.2018.0214 (PMC6284771; doi:10.14336/AD.2018.0214)
Supplement: Supplementary file 1 — Supplementary Data are avalible online at www.aginganddisease.org/EN/10.14336/AD.2018.0214 [file AD-9-6-1058-s.pdf]

SUPPLEMENTARY DATA

MiRNA-10b Reciprocally Stimulates Osteogenesis and Inhibits Adipogenesis Partly through the TGF-β/SMAD2 Signaling Pathway

Hongling Li[1,#], Junfen Fan[1,#], Linyuan Fan[1], Tangping Li[1], Yanlei Yang[1], Haoying Xu[1], Luchan Deng[1], Jing Li[1], Tao Li[2,3], Xisheng Weng[2], Shihua Wang[1,*], Robert Chunhua Zhao[1,*]

[1]Institute of Basic Medical Sciences Chinese Academy of Medical Sciences, School of Basic Medicine Peking Union Medical College, Center of Excellence in Tissue Engineering Chinese Academy of Medical Sciences, Beijing Key Laboratory (No. BZO381), Beijing 100005, China. [2]Department of Orthopaedic Surgery, Peking Union Medical College Hospital, Peking Union Medical College, Beijing 100730, China. [3]Current address: Department of Orthopaedic Surgery, The Affiliated Hospital of Qingdao University, Qingdao 266003, China.

© 2018. Li H et al. Published online at http://www.aginganddisease.org/EN/10.14336/AD.2018.0214

SUPPLEMENTARY DATA

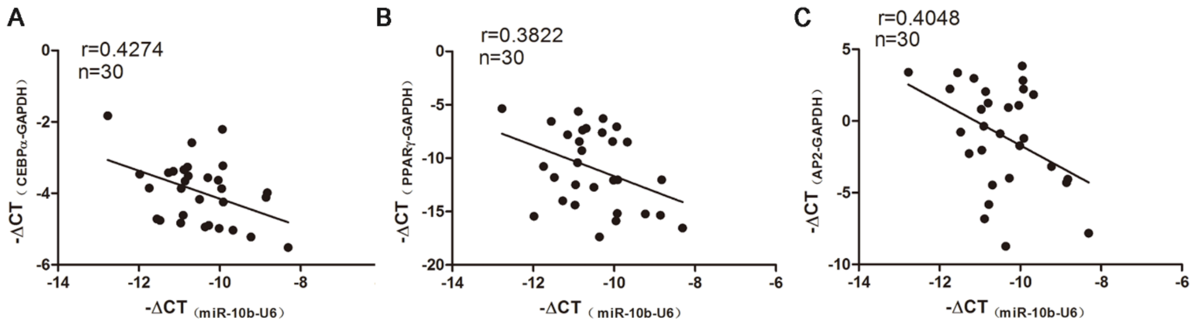

Supplementary Figure 1. The correlation between the expression of adipogenic-related genes and miR-10b in clinical osteoporosis samples. (A) The correlation of miR-10b and CEBPα was analyzed by qRT-PCR. **(B)** The correlation of miR-10b and PPARγ was analyzed by qRT-PCR. **(C)** The correlation of miR-10b and AP2 was analyzed by qRT-PCR. The data, normalized to U6, are averages of 3 independent experiments (mean ± SD).

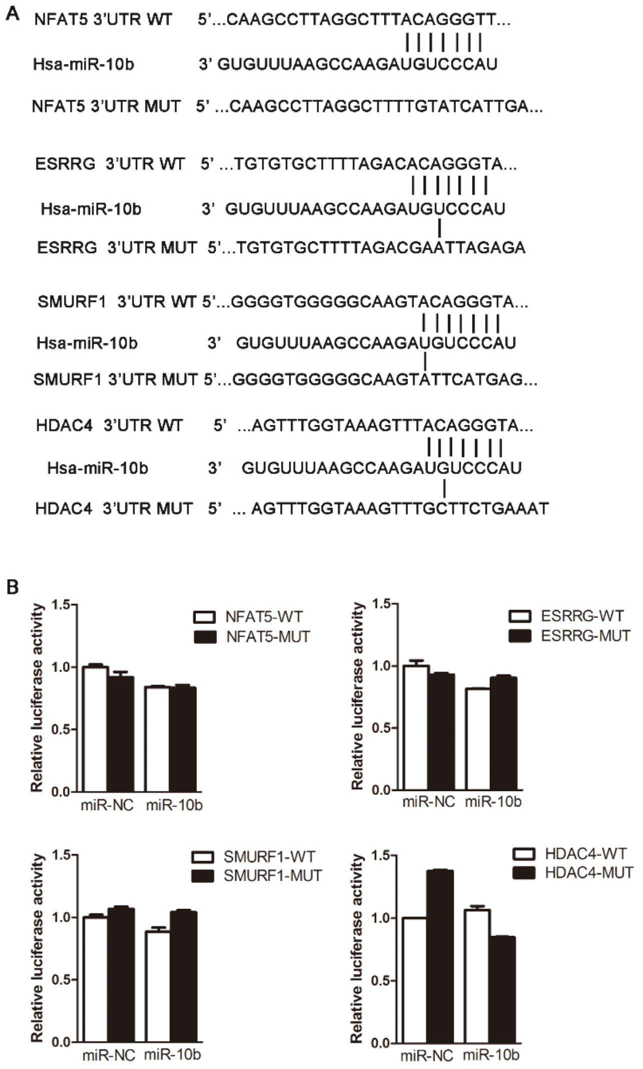

Supplementary Figure 2. NFAT5, ESRRG, SMURF1 and HDAC4 are not the direct targets of miR-10b. (A) Computational analysis was performed to determine the complementarities of the miR-10b seed sequence to the 3'UTRs of NFAT5, ESRRG, SMURF1 and HDAC4. A wild-type (WT) or mutant-type (MUT) construct was inserted into the psiCHECK-2 reporter vector. **(B)** Luciferase activities were measured in the lysates, and the values were normalized to the psiCHECK-2 vector and presented as the fold change of miR-NC.

© 2018. Li H et al. Published online at http://www.aginganddisease.org/EN/10.14336/AD.2018.0214

SUPPLEMENTARY DATA

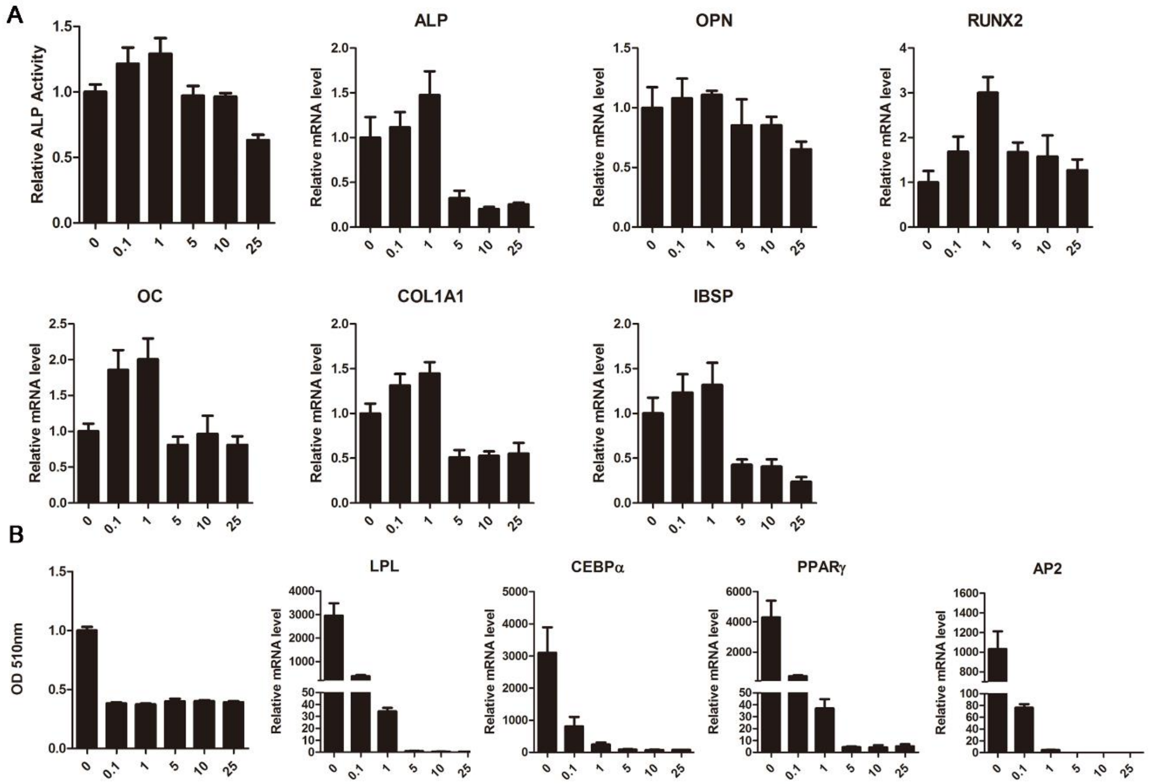

Supplementary Figure 3. The effect of TGF-β1 on osteogenic and adipogenic differentiation. (A) ALP activity and osteoblast marker genes were detected after different concentrations of TGF-β1 treatment. **(B)** Oil red O extraction and adipocyte marker genes were analyzed after different concentrations of TGF-β1 treatment.

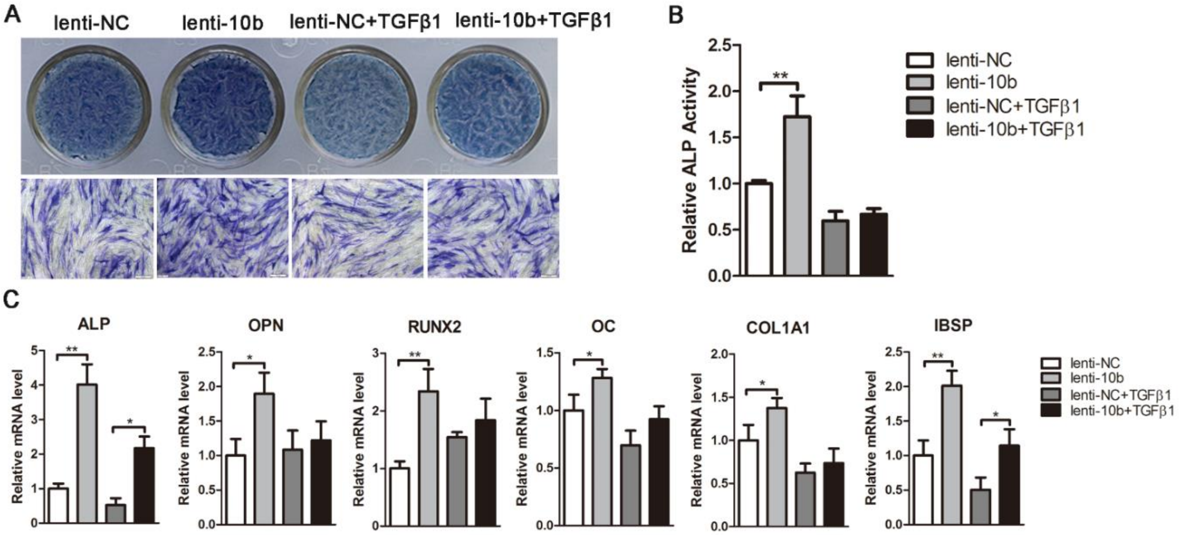

Supplementary Figure 4. 5ng/ml TGF-β1 blocked the enhancement on osteogenic differentiation mediated by miR-10b. (A and **B)** ALP staining and activity were performed on day 4. **(C)** The mRNA levels of osteogenic genes were analyzed by qRT-PCR after different treatments. The data normalized to GAPDH are averages of 3 independent experiments (mean±SD). $^{*}P<0.05$; $^{**}P<0.01$ compared with the control. Scale bars: 200μm.

© 2018. Li H et al. Published online at http://www.aginganddisease.org/EN/10.14336/AD.2018.0214

SUPPLEMENTARY DATA

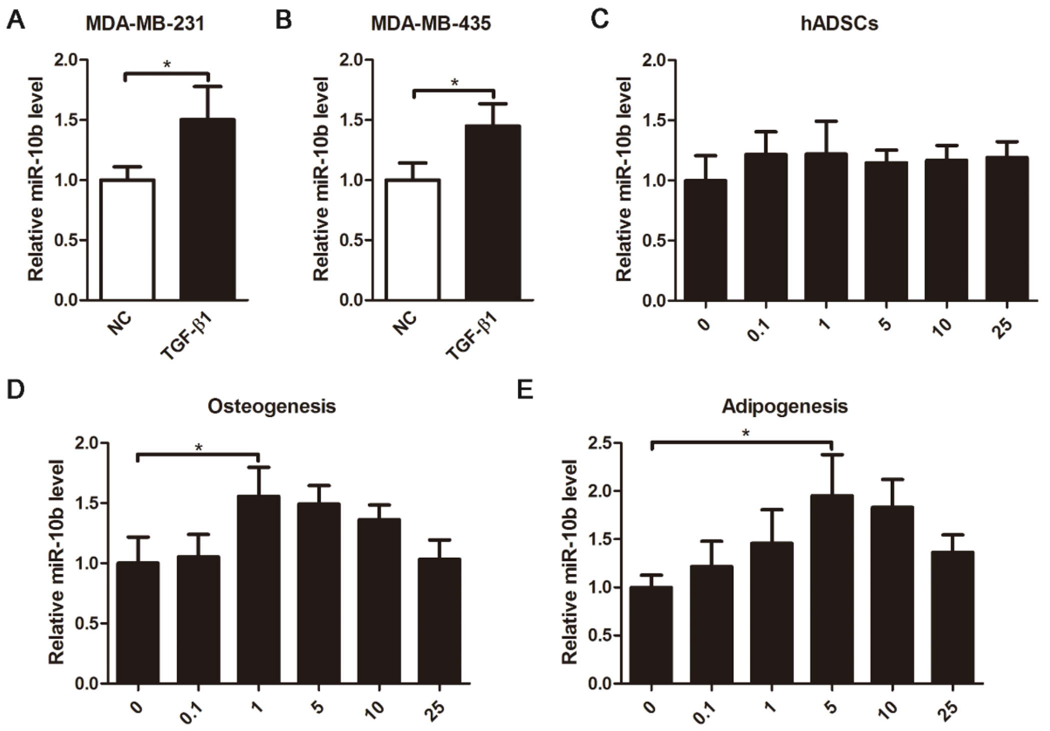

Supplementary Figure 5. miR-10b expression is modulated by TGF-β1 during osteogenesis and adipogenesis of hADSCs. (A) qRT-PCR was used to detect miR-10b expression in MDA-MB-231 cells after TGF-β1 stimulation. **(B)** qRT-PCR was used to detect miR-10b expression in MDA-MB-435 cells after TGF-β1 stimulation. **(C)** The expression level of miR-10b in hADSCs was analyzed after different concentrations of TGF-β1 stimulation. **(D)** The expression level of miR-10b during hADSC osteogenesis was analyzed after different concentrations of TGF-β1 stimulation. **(E)** The expression level of miR-10b during hADSC adipogenesis was analyzed after different concentrations of TGF-β1 stimulation. The data, normalized to GAPDH, are averages of 3 independent experiments (mean±SD). *P<0.05 compared with the control.

© 2018. Li H et al. Published online at http://www.aginganddisease.org/EN/10.14336/AD.2018.0214

SUPPLEMENTARY DATA

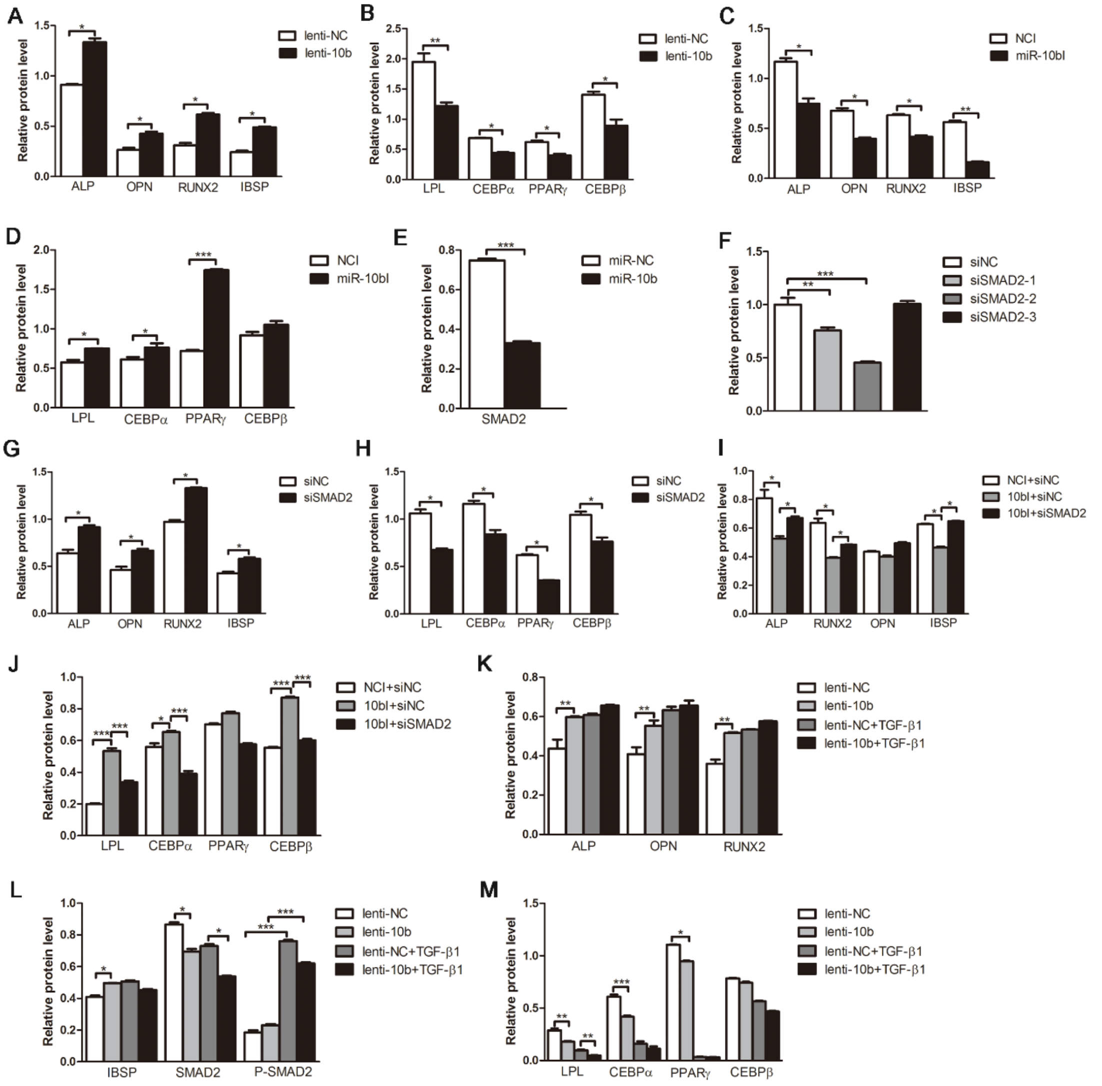

Supplementary Figure 6. The quantitation graphs of western blots. (A) Statistical analysis of the expression of ALP, OPN, RUNX2 and IBSP after miR-10b overexpression (Corresponding to Figure 2F). **(B)** Statistical analysis of the expression of LPL, CEBPα, PPARγ and CEBPβ after miR-10b overexpression (Corresponding to Figure 2J). **(C)** Statistical analysis of the expression of ALP, OPN, RUNX2 and IBSP after miR-10b downregulation (Corresponding to Figure 4F)**. (D)** Statistical analysis of the expression of LPL, CEBPα, PPARγ and CEBPβ after miR-10b downregulation (Corresponding to Figure 4J). **(E)** Statistical analysis of SMAD2 expression in hADSCs (Corresponding to Figure 5C). **(F)** Statistical analysis of the expression of SMAD2 in hADSCs after three SMAD2 siRNAs transfection (Corresponding to Figure 5D). **(G)** Statistical analysis of the expression of osteogenic markers after SMAD2 knockdown (Corresponding to Figure 5H). **(H)** Statistical analysis of adipogenic markers expression after SMAD2 knockdown (Corresponding to Figure 5K). **(I)** Statistical analysis of the expression of osteogenic markers after different treatments (Corresponding to Figure 6C). **(J)** Statistical analysis of the expression of adipogenic markers after different treatments (Corresponding to Figure 6G). **(K)** Statistical analysis of the expression of ALP, OPN and RUNX2 after different treatments (Corresponding to Figure 7E). **(L)** Statistical analysis of the expression of IBSP, SMAD2 and P-SMAD2 after different treatments (Corresponding to Figure 7E). **(M)** Statistical analysis of the expression of adipocyte markers after different treatments (Corresponding to Figure 7E). The data, normalized to GAPDH, are averages of 3 independent experiments (mean±SD). $^{*}P<0.05$; $^{**}P<0.01$; $^{***}P<0.001$ compared with the control.

© 2018. Li H et al. Published online at http://www.aginganddisease.org/EN/10.14336/AD.2018.0214

SUPPLEMENTARY DATA

Supplementary Table 1. The primers of related genes

Gene name	Forward sequence (5'-3')	Reverse sequence (5'-3')
RUNX2	TGTCATGGCGGGTAACGAT	AAGACGGTTATGGTCAAGGTGAA
ALP	CCACGTCTTCACATTTGGTG	AGACTGCGCCTGGTAGTTGT
OPN	ACTCGAACGACTCTGATGATGT	GTCAGGTCTGCGAAACTTCTTA
IBSP	CCCCACCTTTTGGGAAAACCA	TCCCCGTTCTCACTTTCATAGAT
COL1A1	CCCAAGGAAAAGAAGCACGTC	AGGTCAGCTGGATAGCGACATC
OC	GGCAGCGAGGTAGTGAAGA	CCTGAAAGCCGATGTGGT
GAPDH	GGTCACCAGGGCTGCTTTTA	GGATCTCGCTCCTGGAAGATG
PPARγ	CCTATTGACCCAGAAAGCGATT	CATTACGGAGAGATCCACGGA
C/EBPα	AGGAACACGAAGCACGATCAG	CGCACATTCACATTGCACAA
C/EBPβ	CTTCAGCCCGTACCTGGAG	GGAGAGGAAGTCGTGGTGC
LPL	ACAAGAGAGAACCAGACTCCAA	AGGGTAGTTAAACTCCTCCTCC
AP2	AGCACCATAACCTTAGATGGGG	CGTGGAAGTGACGCCTTTCA
SMAD2	CGTCCATCTTGCCATTCACG	CTCAAGCTCATCTAATCGTCCTG
siSMAD2-1	CCAAGCACUUGCUCUGAAATT	UUUCAGAGCAAGUGCUUGGTT
siSMAD2-2	GGUGUUCGAUAGCAUAUUATT	UAAUAUGCUAUCGAACACCTT
siSMAD2-3	CCCUGCAACAGUGUGUAAATT	UUUACACACUGUUGCAGGGTT

RUNX2, runt-related transcription factor 2; ALP, alkaline phosphatase; OPN, osteopontin; IBSP, integrin-binding sialoprotein; OC, osteocalcin; PPARγ, peroxisome proliferator-activated receptor gamma; LPL, lipoprotein lipase; CEBPα/β, CCAAT/enhancer binding protein α/β; AP2, namely FABP4, fatty acid binding protein 4.

Supplementary Table 2. The sequences of fragments in dual luciferase reporter assay

Gene name	Sequence
SMAD2-WT-F	5'-TCGAGAACCCTTAATTATCTTACTTGTCTATGAAGGAGATAAACAG GGTACTGTACTGGAGAATAACAGATGGGATGCGC-3'
SMAD2-WT-R	5'-GGCCGCGCATCCCATCTGTTATTCTCCAGTACAGTACCCTGTTTATCT CCTTCATAGACAAGTAAGATAATTAAGGGTTC-3'
SMAD2-MUT-F	5'-TCGAGAACCCTTAATTATCTTACTTGTCTATGAAGGAGATAACTGTAC TGGAGAATAACAGATGGGATGCGC-3'
SMAD2-MUT-R	5'-GGCCGCGCATCCCATCTGTTATTCTCCAGTACAGTTATCTCCTTCATAG ACAAGTAAGATAATTAAGGGTTC-3'
ESRRG-WT-F	5'-TCGAGGTGTTTGGATTCTTGTGGTGTGTGCTTTTAGACACAGGGTAGA ATTAGAGACAATATTGGATGTACAATTCCTCAGG-3'
ESRRG-WT-R	5'-GGCCGCCTGAGGAATTGTACATCCAATATTGTCTCTAATTCTACCCTGT GTCTAAAAGCACACACCACAAGAATCCAAACACC-3'
ESRRG-MUT-F	5'-TCGAGGTGTTTGGATTCTTGTGGTGTGTGCTTTTAGACGAATTAGAGA CAATATTGGATGTACAATTCCTCAGGC-3'

© 2018. Li H et al. Published online at http://www.aginganddisease.org/EN/10.14336/AD.2018.0214

ESRRG-MUT-R	5’-GGCCGCCTGAGGAATTGTACATCCAATATTGTCTCTAATTCGTCTAAAAGCACACACCACAAGAATCCAAACACC-3’
NFAT5-WT-F	5’-TCGAGCTTACCTGATGGTTCCCACAAGCCTTAGGCTTTACAGGGTTGTATCATTGACTTAAAATGAAGAATTAACTTGTGGC-3’
NFAT5-WT-R	5’-GGCCGCCACAAGTTAATTCTTCATTTTAAGTCAATGATACAACCCTGTAAAGCCTAAGGCTTGTGGGAACCATCAGGTAAGC-3’
NFAT5-MUT-F	5’-TCGAGCTTACCTGATGGTTCCCACAAGCCTTAGGCTTTTGTATCATTGACTTAAAATGAAGAATTAACTTGTGGC-3’
NFAT5-MUT-R	5’-GGCCGCCACAAGTTAATTCTTCATTTTAAGTCAATGATACAAAAGCCTAAGGCTTGTGGGAACCATCAGGTAAGC-3’
SMURF1-WT-F	5’-TCGAGGTTTCTTTGTTGTTGTTTTTGTTTGGGGTGGGGGCAAGTACAGGGTAATTCATGAGCAAGACATTTCACTGCTGTGC- 3’
SMURF1-WT-R	5’-GGCCGCACAGCAGTGAAATGTCTTGCTCATGAATTACCCTGTACTTGCCCCCACCCCAAACAAAAACAACAACAAAGAAACC-3’
SMURF1-MUT-F	5’-TCGAGGTTTCTTTGTTGTTGTTTTTGTTTGGGGTGGGGGCAAGTATTCATGAGCAAGACATTTCACTGCTGTGC-3’
SMURF1-MUT-R	5’-GGCCGCACAGCAGTGAAATGTCTTGCTCATGAATACTTGCCCCCACCCCAAACAAAAACAACAACAAAGAAACC-3’
HDAC4-WT-F	5’-TCGAGGACCCAGAGCCGAGTTTCGGAGTTTGGTAAAGTTTACAGGGTAGCTTCTGAAATTAACTCAAACTTTTGACCAAATGAGC-3’
HDAC4-WT-R	5’-GGCCGCTCATTTGGTCAAAAGTTTGAGTTAATTTCAGAAGCTACCCTGTAAACTTTACCAAACTCCGAAACTCGGCTCTGGGTCC-3’
HDAC4-MUT-F	5’-TCGAGGACCCAGAGCCGAGTTTCGGAGTTTGGTAAAGTTTGCTTCTGAAATTAACTCAAACTTTTGACCAAATGAGC-3’
HDAC4-MUT-R	5’-GGCCGCTCATTTGGTCAAAAGTTTGAGTTAATTTCAGAAGCAAACTTTACCAAACTCCGAAACTCGGCTCTGGGTCC-3’

© 2018. Li H et al. Published online at http://www.aginganddisease.org/EN/10.14336/AD.2018.0214
